# Structural Changes of Gut Microbiota during Berberine-Mediated Prevention of Obesity and Insulin Resistance in High-Fat Diet-Fed Rats

**DOI:** 10.1371/journal.pone.0042529

**Published:** 2012-08-03

**Authors:** Xu Zhang, Yufeng Zhao, Menghui Zhang, Xiaoyan Pang, Jia Xu, Chaoying Kang, Meng Li, Chenhong Zhang, Zhiguo Zhang, Yifei Zhang, Xiaoying Li, Guang Ning, Liping Zhao

**Affiliations:** 1 State Key Laboratory of Microbial Metabolism, School of Life Sciences and Biotechnology, Shanghai Jiao Tong University, Shanghai, PR China; 2 Shanghai Center for Systems Biomedicine, Shanghai Jiao Tong University, Shanghai, PR China; 3 Shanghai Clinical Center for Endocrine and Metabolic Diseases and Division of Endocrine and Metabolic Diseases, Rui Jin Hospital, Shanghai Jiao Tong University, Shanghai, PR China; Instutite of Agrochemistry and Food Technology, Spain

## Abstract

Berberine, a major pharmacological component of the Chinese herb *Coptis chinensis*, which was originally used to treat bacterial diarrhea, has recently been demonstrated to be clinically effective in alleviating type 2 diabetes. In this study, we revealed that berberine effectively prevented the development of obesity and insulin resistance in high-fat diet (HFD)-fed rats, which showed decreased food intake. Increases in the levels of serum lipopolysaccharide-binding protein, monocyte chemoattractant protein-1, and leptin and decrease in the serum level of adiponectin corrected for body fat in HFD-fed rats were also significantly retarded by the co-administration of berberine at 100 mg/kg body weight. Bar-coded pyrosequencing of the V3 region of 16S rRNA genes revealed a significant reduction in the gut microbiota diversity of berberine-treated rats. UniFrac principal coordinates analysis revealed a marked shift of the gut microbiota structure in berberine-treated rats away from that of the controls. Redundancy analysis identified 268 berberine-responding operational taxonomic units (OTUs), most of which were essentially eliminated, whereas a few putative short-chain fatty acid (SCFA)-producing bacteria, including *Blautia* and *Allobaculum*, were selectively enriched, along with elevations of fecal SCFA concentrations. Partial least square regression models based on these 268 OTUs were established (Q^2^>0.6) for predicting the adiposity index, body weight, leptin and adiponectin corrected for body fat, indicating that these discrete phylotypes might have a close association with the host metabolic phenotypes. Taken together, our findings suggest that the prevention of obesity and insulin resistance by berberine in HFD-fed rats is at least partially mediated by structural modulation of the gut microbiota, which may help to alleviate inflammation by reducing the exogenous antigen load in the host and elevating SCFA levels in the intestine.

## Introduction

Gut microbiota, one of the largest and most populated microbial ecosystems on Earth, has been considered a forgotten organ due to its long neglected roles in human nutrition, metabolism, and immunity [1], [2], [3]. Accumulating evidence indicates that gut microbiota might be associated with the etiology or development of obesity [4], [5] and diabetes [6], [7]. On the one hand, the gut microbiota help to digest otherwise indigestible food components and regulate host fat storage genes, thus modulating host energy homeostasis [5], [8]. On the other hand, imbalances in the structure of the gut microbiota induced by high-fat diet (HFD) consumption may impair gut barrier function and increase the levels of endotoxin in circulating systems, which provokes metabolic endotoxemia and induces insulin resistance, obesity, and even diabetes [7], [9]. Selective modulation of the structure and/or activity of the gut microbiota by using prebiotics or probiotics has been demonstrated to confer beneficial effects in both human and animal trials [10]; for example, Cani *et al*. showed that oral administration of inulin-type fructans significantly increased the abundance of *Bifidobacterium* spp., which essentially prevented HFD-induced obesity in mice [11]. Therefore, the gut microbiota represents a potential target of therapeutic drugs or nutritional interventions [12], [13].

Berberine, an isoquinoline alkaloid, is the major pharmacological component of the Chinese herb *Coptis chinensis* (Huang-Lian, a common herb in traditional Chinese medicine) [14]. As a botanic drug, berberine or berberine-containing herbs have been used to treat intestinal infections, particularly bacterial diarrhea, for thousands of years in China [14]. Recently, we and others demonstrated that berberine was clinically effective in alleviating type 2 diabetes, as it significantly decreased fasting plasma glucose (FBG), postprandial blood glucose, glycated hemoglobin, total cholesterol, and low-density lipoprotein cholesterol levels [15], [16], [17]. The proposed mechanisms of action of berberine include upregulation of hepatic low-density lipoprotein receptor mRNA expression [18], activation of AMP-activated protein kinase in both adipose and muscle tissues [19], stimulation of glycolysis in peripheral tissue cells [20], promotion of insulin secretion [21], inhibition of liver gluconeogenesis [22], and promotion of intestinal glucagon-like protein-1 secretion [23]. However, a paradox remains regarding the mode of action of berberine due to its poor oral bioavailability. It has been reported that the maximum concentration (*C*_max_) of berberine in plasma was 4 ng/ml after the oral administration of 100 mg/kg berberine in rats [24], and a *C*_max_ of 0.4 ng/ml after a single oral dose of 400 mg of berberine has been reported in humans [25]. Effective concentrations (at the level of micrograms per milliliter) required for *in vitro* assays cannot be achieved as a result of this low bioavailability; for example, a concentration exceeding 2.5 µg/ml was needed to upregulate low-density lipoprotein receptor mRNA expression in HepG2 cells [18]. Therefore, we cannot fully explain the clinical efficacy of berberine in patients because the primary target by which berberine regulates the aforementioned genes or pathways is largely unknown.

As berberine is poorly absorbed into the bloodstream from the gut, modulation of gut microbiota has been hypothesized as one of the mechanisms of its anti-diabetic effect [26], [27], similarly to many other traditional Chinese herbal medicines [28], [29]. It has been reported that berberine significantly decreased the relative abundances of both Firmicutes and Bacteroidetes in the gut of HFD-fed mice, increased fasting-induced adipose factor (*Fiaf*) gene expression in both intestinal and adipose tissues, indicating that the antimicrobial activity of berberine may contribute to its anti-obesity effects [27]. *Lactobacillus* sp. (CICC21024), a species of Firmicutes, was also found to be significantly inhibited by berberine *in vitro*. In another previous report by Chae *et al.* [30], however, weak or no inhibition by berberine was observed against two species of *Lactobacillus*. Selective inhibition of specific species of bacteria in the same genus was also observed in the case of *Bifidobacterium* by Chae *et al.* [30], which indicates that the modulation of gut microbiota by berberine may occur at the species level. In this study, we assessed the preventive effects of berberine on HFD-induced obesity and insulin resistance in rats, resolved the species-level structural changes of gut microbiota, and identified species-level phylotypes which are associated with host phenotypes by using a microbiome-wide association study (MiWAS) strategy [31].

## Results

### Berberine-mediated prevention of obesity in HFD-fed rats

**Figure 1:**
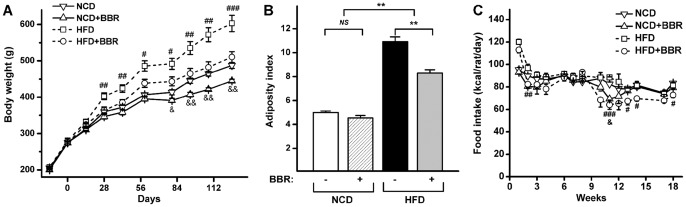
Effects of berberine on body weight gain, the adiposity index and food intake in HFD-fed rats. (A) Body weight gain; (B) adiposity index, calculated as the fat pad weight (sum of epididymal and perirenal fat pads) per 100 g of total body weight; (C) food intake. Values are expressed as means ± standard error. Differences were assessed by ANOVA and denoted as follows: ^#^ P<0.05, ^##^ P<0.01, ^###^ P<0.001, HFD vs. HFD+BBR; ^&^ P<0.05, ^&&^ P<0.01, NCD vs. NCD+BBR; ^**^ P<0.01; *^NS^* not significant.

Compared with normal chow diet (NCD)-fed rats, HFD feeding over 4 weeks induced a significant increase in the body weight of rats (Figure 1A). Co-administration of berberine at a dose of 100 mg/kg body weight prevented the body weight increase observed in HFD-fed rats; in fact, animals in this group (HFD+BBR group) displayed no significant difference in body weight from those in the NCD group during the entire trial. Berberine also tended to reduce the body weight of NCD-fed rats, albeit to a lesser extent than that in HFD-fed rats (Figure 1A). The food intake of HFD-fed rats was reduced by berberine, particularly in the last 8 weeks, whereas smaller decrease in food intake was observed in NCD-fed rats (Figure 1C). The adiposity index was calculated as body fat weight (represented as the sum of epididymal and perirenal fat pads) per 100 g total body weight [32]. Berberine significantly decreased the adiposity index in HFD-fed rats but not in NCD-fed rats (Figure 1B).

### Berberine-mediated preservation of insulin sensitivity in HFD-fed rats

**Figure 2:**
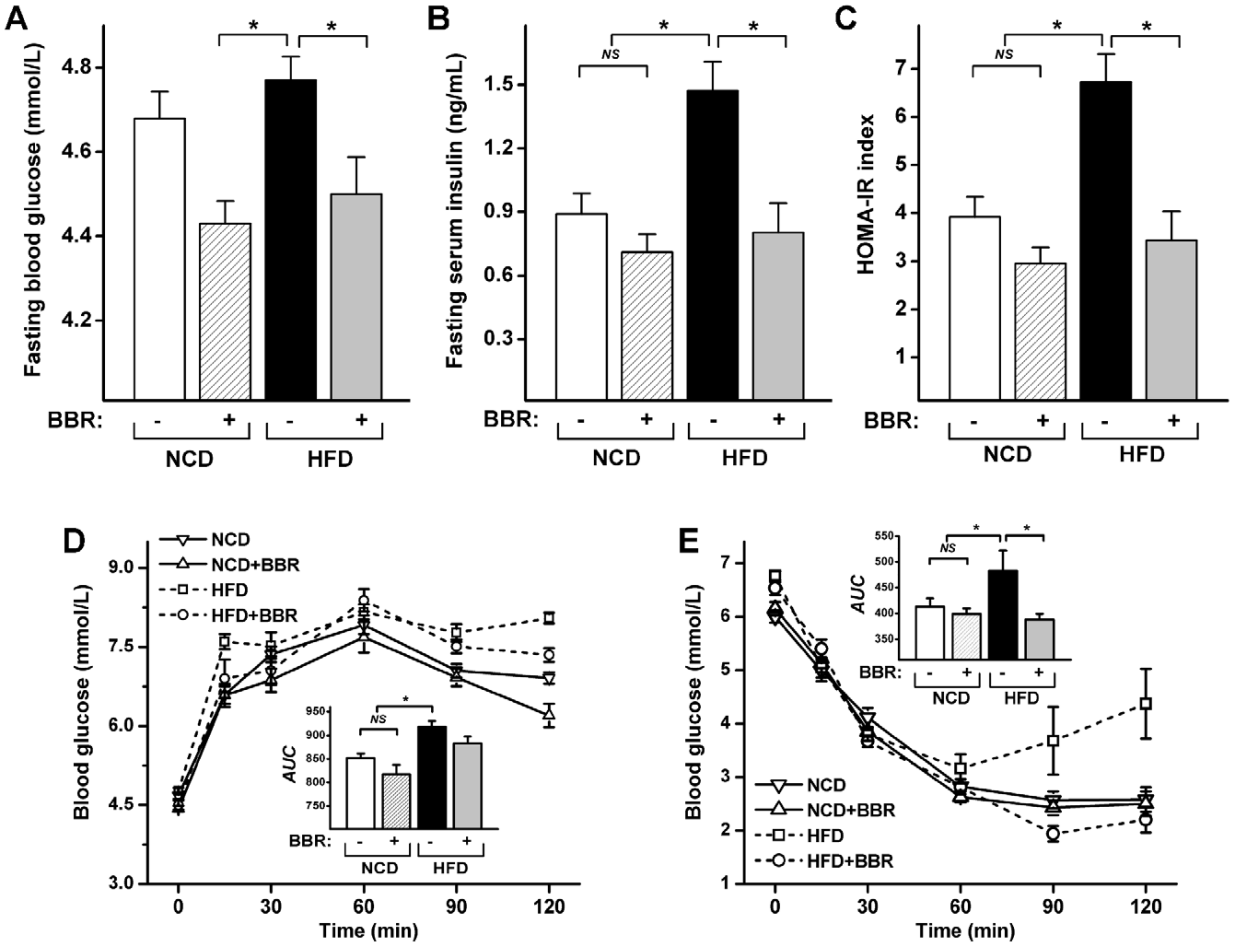
Preventive effects of berberine on the development of insulin resistance induced by HFD feeding in rats. (A) FBG; (B) FINS; (C) HOMA-IR, calculated according to the formula fasting insulin (µU/mL)×fasting glucose (mmol/L)/22.5; (D) OGTT test; (E) ITT test. Curves of blood glucose levels and the calculated *AUC* (inner graph) are shown. Values are expressed as means ± standard error. Differences were assessed by ANOVA and denoted as follows: ^*^ P<0.05; *^NS^* not significant.

Although there was no significant difference in FBG levels between the NCD and HFD groups, HFD-fed rats exhibited significantly elevated fasting insulin (FINS) and homeostasis assessment of insulin resistance (HOMA-IR) values (Figure 2A, B, and C). Results of the oral glucose tolerance test (OGTT) and intraperitoneal insulin tolerance test (ITT) confirmed the impaired glucose and insulin tolerance in HFD-fed rats (Figure 2D and E). Berberine administration prevented the increases of FBG, FINS, HOMA-IR, and area under the curve of the ITT test (*AUC*_ITT test_). The area under the curve of the OGTT test (*AUC*_OGTT test_) exhibited the same trend, but the difference did not reach significance. Similar effects of berberine were also observed in NCD-fed rats, although not to statistically significant levels (Figure 2).

### Effects of berberine on systemic inflammation in HFD-fed rats

**Figure 3:**
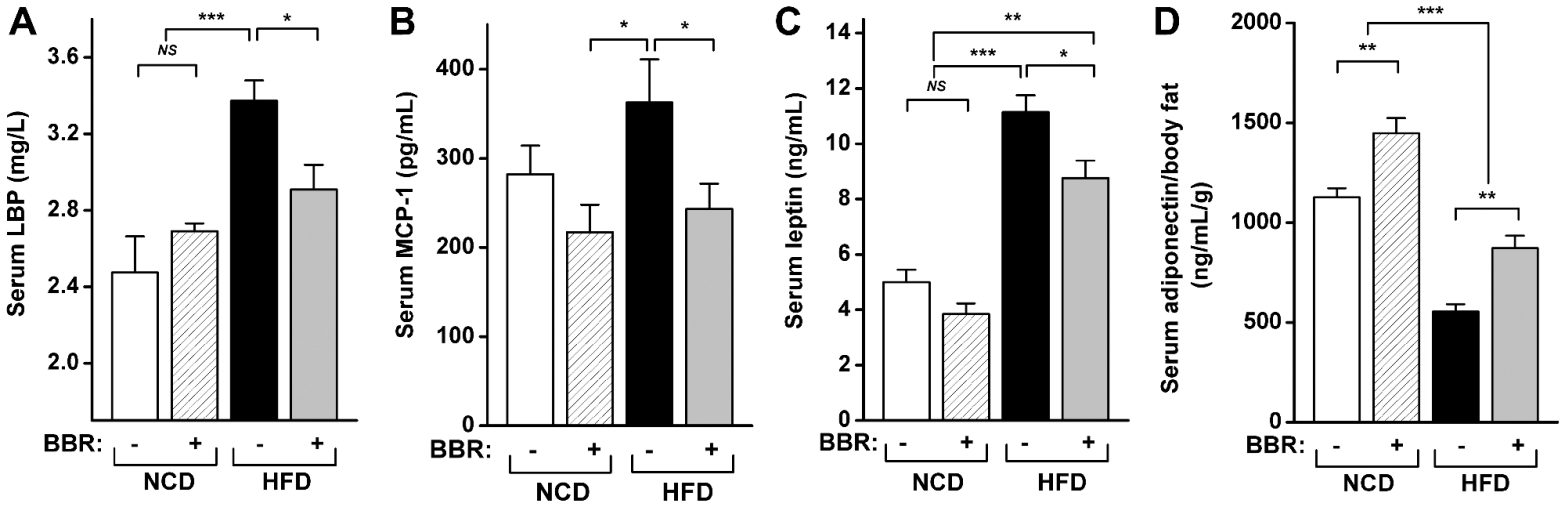
Influences of berberine on systemic inflammation in HFD-fed rats. (A) Serum LBP; (B) serum leptin; (C) serum MCP-1; (D) serum adiponectin corrected for body fat. Values are expressed as means ± standard error. Differences were assessed by ANOVA and denoted as follows: ^*^ P<0.05; ^**^ P<0.01; ^***^ P<0.001; *^NS^* not significant.

Lipopolysaccharide (LPS)-binding protein (LBP), monocyte chemoattractant protein-1 (MCP-1), leptin, and adiponectin were measured at the end of the trial. Rats in the HFD group had a significantly higher serum level of LBP, a biomarker of circulating exogenous antigen [33], and this increase was essentially prevented by berberine co-administration (P<0.05, Figure 3A). Using MCP-1 as an indicator of inflammation [34], we demonstrated that HFD consumption increased inflammation, which was effectively reduced to the level observed in NCD-fed rats by berberine (P<0.05, Figure 3B). Serum levels of leptin were also elevated by HFD feeding and prevented by berberine co-administration (P<0.05, Figure 3C). Serum concentrations of adiponectin were higher in HFD-fed rats than in NCD-fed rats (data not shown); however, when corrected for body fat weight [35], [36], the serum concentrations of adiponectin were significantly lower in HFD-fed rats than in NCD-fed rats (P<0.001, Figure 3D). The co-administration of berberine significantly prevented the decrease in serum adiponectin levels corrected for body fat in HFD-fed rats (P<0.01, Figure 3D).

### Overall structural changes of the gut microbiota in response to berberine treatment

A total of 287,700 usable pyrosequencing reads (53,865 unique sequences) were obtained for 120 samples. After discarding sequences that had no near-neighbors in the entire Greengenes database, 287,176 reads (average of 2393 sequences per sample) were delineated into 6720 operational taxonomic units (OTUs) at the 98% similarity level with Distance-Based OTU and Richness (DOTUR). Rarefaction and Shannon diversity curves revealed that although new rare phylotypes would be expected with additional sequencing, most of the diversity had already been captured (Figure S1). Approximately half of the 6720 OTUs (3140 OTUs) existed in a single sample but only contributed to 1.33% of all reads. A total of 6082 OTUs (contributing to 96.5% of all sequencing reads) were assigned to defined phyla by RDP classifier with a bootstrap cutoff of 50%. The most abundant phyla included Firmicutes (3764 OTUs, contributing to 53.51% of all reads), Bacteroidetes (1753 OTUs, contributing to 34.83% of all reads), Proteobacteria (341 OTUs, contributing to 6.22% of all reads), and Actinobacteria (139 OTUs, contributing to 1.15% of all reads). As revealed by taxon-based analysis, significantly higher abundances of the phyla Actinobacteria and Verrucomicrobia were observed in the HFD group than in the NCD group (P<0.05), and these higher abundances were completely reverted by berberine co-administration (Table S1, Materials and Methods S1). Additionally, TM7 was inhibited by berberine in both HFD- and NCD-fed rats. No significant difference was observed in the relative abundances of Firmicutes, Bacteroidetes, and Proteobacteria and the ratio of Firmicutes to Bacteroidetes among the groups in this study (Table S1, Materials and Methods S1).

**Figure 4:**
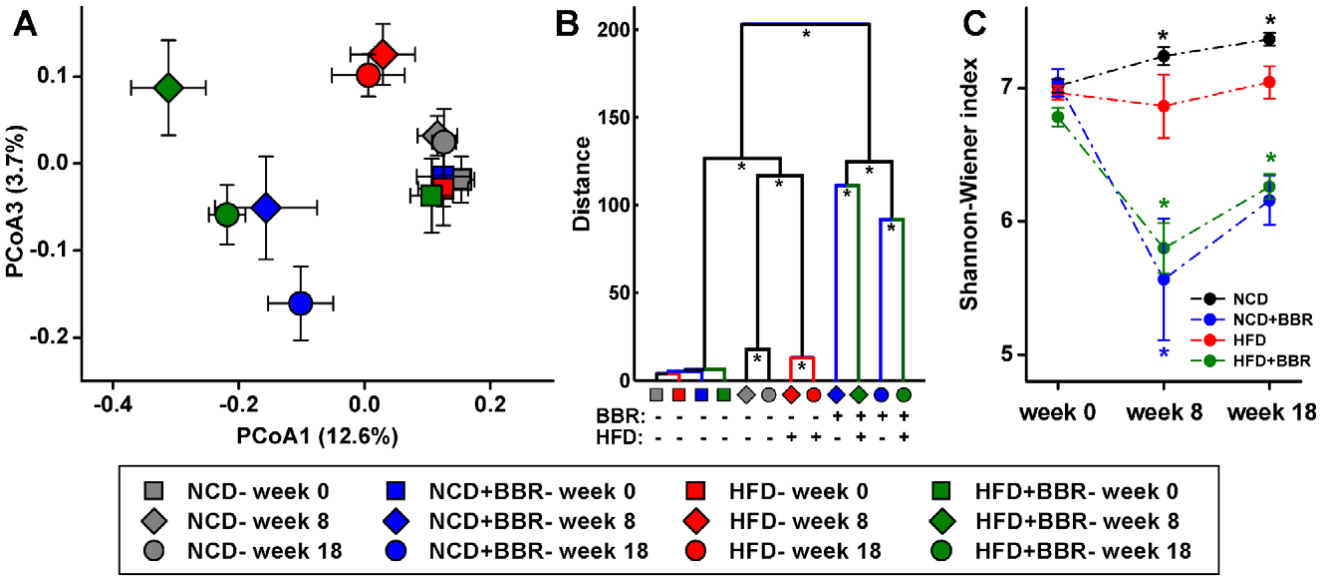
Overall structural changes of gut microbiota. (A) PCoA score plot based on unweighted UniFrac metrics. Each point represents the mean principal component scores of all rats in a group at one time point, and the error bar represents the standard deviation. (B) Clustering of gut microbiota based on mahalanobis distances calculated using MANOVA, ^*^ P<0.05. (C) Shannon-Wiener index, calculated after rarefying to an equal number of sequence reads for all samples. Values are expressed as means ± standard error. ^*^ P<0.05, significant difference when compared with the 0^th^ week value as assessed by ANOVA.

Unweighted UniFrac principal coordinates analysis (PCoA) revealed that the gut microbiota structure changed significantly in response to HFD feeding and berberine administration. Berberine-related differences were mainly observed along the first principal coordinate (PCoA1), which accounted for the largest proportion (12.6%) of total variation (Figure 4A). This was confirmed by multivariate analysis of variance (MANOVA), which first separated animals into two clusters corresponding to groups treated with or without berberine (Figure 4B). HFD also contributed to significant variation in the gut microbiota structure, which mainly distributed along the third principal coordinate (PCoA3). As shown in Figure 4A, berberine, on the one hand, drove the gut microbiota changes along PCoA1, and on the other hand, partly reversed HFD-induced variation along PCoA3. Similar patterns were observed in principal component analysis (PCA; Figure S2A and B). In the case of weighted UniFrac PCoA, no sharp separation was observed, although the largest variation was also explained by the treatment of berberine as revealed by MANOVA (Figure S2C and D).

The Shannon-Wiener diversity index revealed that berberine significantly decreased the bacterial diversity of the gut microbiota in both NCD- and HFD-fed rats (Figure 4C). This was confirmed by two other diversity indices: Simpson's diversity index and Buzas and Gibson's evenness (Figure S3A and B). The richness of the gut microbiota was also significantly reduced by berberine, as revealed by rarefaction and Chao1 estimates (Figure S3C and D). Total bacterial quantification with real-time polymerase chain reaction (RT-PCR) demonstrated that there was no significant difference in the total bacterial population among rats in the NCD, NCD+BBR, and HFD groups. However, a significantly reduced total bacterial population was observed in the HFD+BBR group compared with those in the other groups (P<0.05, Figure S4, Materials and Methods S1).

### Key phylotypes of the gut microbiota responding to berberine treatment in rats

**Figure 5:**
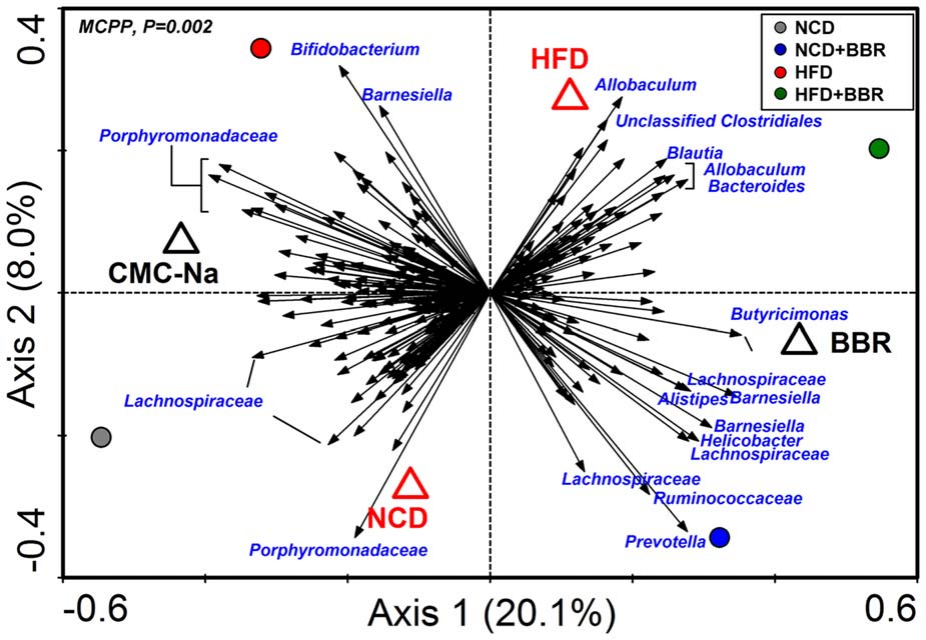
Distance triplot of the RDA of gut microbiota. Nominal environmental variables (BBR, CMC-Na, HFD, and NCD) are indicated by open triangles. Samples are indicated by filled circles. A total of 268 OTUs that had at least 20% of the variability in their values explained by the first axis are indicated by black arrows. Relative better-fitting species are labeled with taxonomic names (genus or family names). Upper left, P-value obtained by MCPP. BBR, berberine; CMC-Na, sodium carboxymethylcellulose as a vehicle control; HFD, high-fat diet; NCD, normal chow diet.

**Figure 6:**
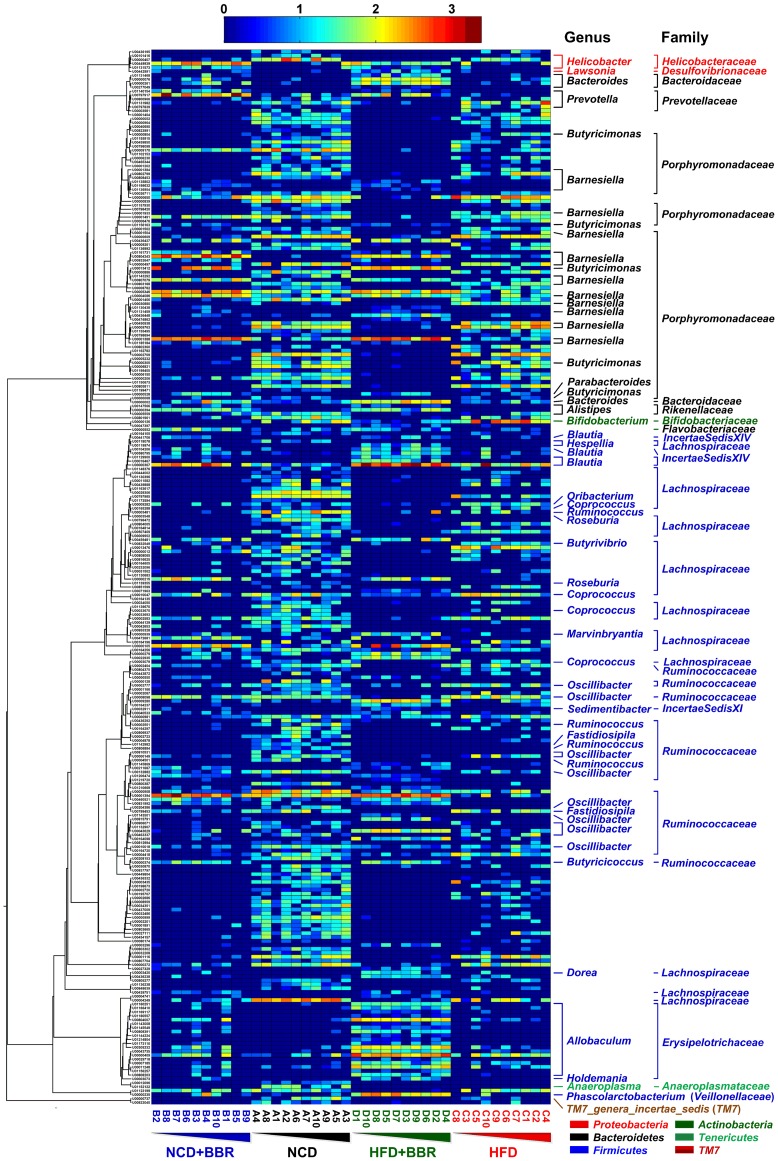
Heat map of RDA-identified key OTUs responding to berberine treatment. The color of the spot corresponds to the normalized and log-transformed relative abundance of the OTU. The OTUs are organized according to their phylogenetic positions. The family and genus names of the OTUs are shown on the right. Samples in each group were organized according to the adiposity index (ascending order).

To identify key phylotypes of the gut microbiota responding to berberine treatment, redundancy analysis (RDA) was used to analyze the pyrosequencing data of the samples in the 18^th^ week. The major difference in the gut microbiota structure corresponded to berberine treatment along the first ordination axis, explaining 20.1% of the total variability (Figure 5). Both berberine administration and HFD feeding led to significant changes in the gut microbiota structure, as validated by the Monte Carlo permutation procedure (MCPP; P = 0.002). We identified 268 key OTUs that had at least 20% of the variability in their values explained by the first axis, most of which distributed across such families as *Porphyromonadaceae* (65 OTUs), *Lachnospiraceae* (50 OTUs), *Ruminococcaceae* (38 OTUs), *Erysipelotrichaceae* (20 OTUs), *Prevotellaceae* (6 OTUs), *Incertae Sedis XIV* (5 OTUs), *Helicobacteraceae* (4 OTUs), and *Rikenellaceae* (3 OTUs) (Figure 6 and Table S2).

In total, 174 of the 268 identified key OTUs were eliminated or decreased by berberine, whereas the remaining 94 OTUs were enriched. All identified OTUs belonging to *Allobaculum* (19 OTUs) and *Blautia* (5 OTUs) were markedly enriched by berberine, particularly under HFD feeding (Figure 6 and Table S3). Taxon-based analysis at the genus level also demonstrated that the relative abundance of *Allobaculum* and *Blautia* was significantly higher in the HFD+BBR group than in the HFD group (P<0.01, with median abundances of 9.07% vs. 1.6% and 7.35% vs. 0.76%, respectively) (Table S1, Materials and Methods S1).

Other relatively abundant OTUs that were enriched by berberine included those belonging to *Bacteroides* (3 OTUs), *Butyricimonas* (1 OTU), *Phascolarctobacterium* (1 OTU), *Prevotella* (1 OTU), unclassified *Porphyromonadaceae* (5 OTUs), and unclassified *Ruminococcaceae* (2 OTU). *Barnesiella* is an important genus that is significantly influenced by berberine, albeit with different behaviors among OTUs. Twenty-one OTUs belonging to this genus were identified, 10 of which were enriched by berberine, whereas the remaining 11 OTUs were decreased (Figure 6 and Table S3). The most abundant OTU (U00000126) in the genus *Bifidobacterium* exhibited a significantly higher median abundance in the HFD group (1.77%) which was completely reverted by berberine to a level similar to that in the NCD group (with median abundances of 0.04% vs. 0.12%, P>0.05) (Table S3).

**Figure 7:**
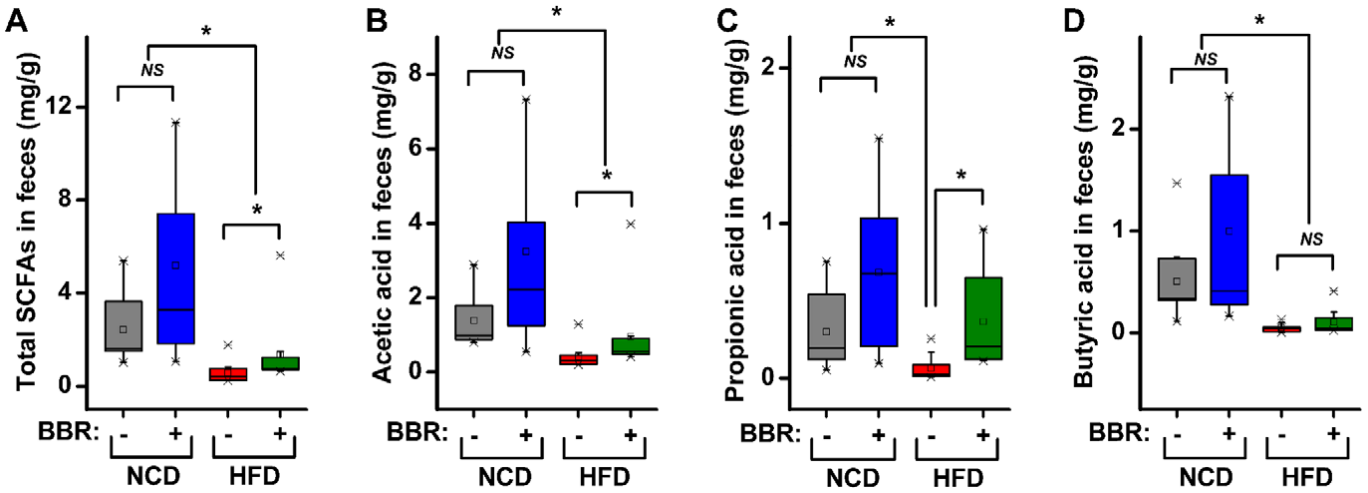
Fecal concentrations of SCFAs in rats. Levels of total SCFAs (A), acetic acid (B), propionic acid (C), and butyric acid (D) are shown. Total SCFA levels were calculated as the sum of acetic acid, propionic acid, butyric acid, *n*-valeric acid, isobutyric acid, and isovaleric acid. The median (central thick lines), 25% and 75% quartile ranges (box width), and upper and lower limits (error bar) of each group are shown in the box plot. Differences were assessed by the Mann-Whitney test, ^*^ P<0.05; *^NS^* not significant.

As most members in *Allobaculum* and *Blautia* were short-chain fatty acid (SCFA) producers, we determined the fecal concentration of SCFAs, including acetic acid, propionic acid, butyric acid, *n*-valeric acid, isobutyric acid, and isovaleric acid, in the animals by gas chromatography. The results indicated that berberine administration significantly increased the fecal concentration of total SCFAs, particularly acetic acid and propionic acid, in HFD-fed rats (Figure 7A, B, and C). Butyric acid exhibited the same trend, but the difference did not reach significance (Figure 7D).

### Prediction of host metabolic phenotypes via partial least squares (PLS) regression modeling based on the RDA-identified key OTUs

As revealed by the aforementioned results, on the one hand, berberine significantly prevented the development of obesity and insulin resistance induced by HFD. On the other hand, berberine markedly altered the composition of the gut microbiota in rats. To assess whether there is a possible association between the structural changes of the gut microbiota induced by berberine and host phenotype variations, the 268 key OTUs selected above were employed to predict the host phenotypes by using PLS regression models (Table 1). As indicated by the goodness of prediction (Q^2^), most PLS regression models performed well in correlating the identified key OTUs with the host phenotypes, particularly with the adiposity index (Q^2^ = 0.81), serum adiponectin levels corrected for body fat (Q^2^ = 0.67), body weight (Q^2^ = 0.67), and serum leptin levels (Q^2^ = 0.64) (Table 1). The high predictabilities of the models were also confirmed by the Pearson's correlation analysis between the predicted and observed host phenotype values, particularly for the adiposity index (R = 0.91), serum adiponectin levels corrected for body fat (R = −0.84), body weight (R = 0.82), and serum leptin levels (R = 0.81) (Table 1).

**Table 1:** Prediction of host phenotypes using the RDA-identified key OTUs with PLS regression models.

	Q^2^ a	RMSECV^b^	Observed value^c^	R^d^	P^d^
Adiposity index	0.81	1.2	7.19±2.76	0.91	<0.0001
Adiponectin/body fat (ng/mL/g)^e^	0.67	203.7	1000.29±376.50	−0.84	<0.0001
Body weight (g)	0.67	41.52	511.45±71.77	0.82	<0.0001
Leptin (ng/mL)	0.64	2009.03	7188.2±3367.16	0.81	<0.0001
FBG (mmol/L)	0.48	0.2	4.62±0.27	0.7	<0.0001
*AUC*_OGTT test_	0.47	41.88	867.84±57.44	0.69	<0.0001
HOMA-IR	0.44	1.59	4.25±2.12	0.67	<0.0001
MCP-1 (pg/mL)	0.43	96.37	271.02±127.2	0.66	<0.0001
FINS (ng/mL)	0.41	0.36	0.97±0.47	0.64	<0.0001
*AUC*_ITT test_	0.4	60.76	420.64±78.23	0.68	<0.0001
LBP (mg/L)	0.37	409.23	2862.12±513.94	0.62	<0.0001

a Goodness of prediction based on leave-one-out cross-validated PLS regression.

b Root-mean-square error of cross-validation.

c Values observed in the experiment are expressed as means ± standard derivation.

d Pearson's correlation coefficient (R) and P-values.

e Serum adiponectin levels corrected for body fat.

## Discussion

Due to the evident clinical therapeutic effects of berberine for diabetes and dyslipidemia [15], [18], the mechanisms involved in its beneficial effects against metabolic disorders have attracted much attention in recent years. In this study, we demonstrated that berberine co-administration at a dose of 100 mg/kg body weight effectively prevented the weight gain and development of insulin resistance induced by long-term HFD feeding. No obvious side effects were observed in the present study. As extrapolated using the body surface area normalization method [37], such dose is equivalent to about 1.0 g/day/person in humans. In a previous human trial, we also confirmed that oral administration of berberine at such a dose of 1.0 g/day/person for 3 months is effective and safe in the treatment of type 2 diabetes and dyslipidemia [15].

Improvement of insulin sensitivity has been widely reported to be involved in the mechanisms of action of berberine in both animal and human studies. Lee *et al*. [19] reported that berberine alleviated insulin resistance in both *db/db* mice and HFD-fed rats, along with downregulating lipogenic genes and upregulating genes involved in energy expenditure. Kong *et al*. [38] also suggested that berberine restored the impaired insulin sensitivity in rats with type 2 diabetes via a mechanism of protein kinase C-dependent elevation of insulin receptor gene expression. Clinical research further confirmed that the mean percentage of peripheral blood lymphocytes that express insulin receptor on their surface was significantly elevated by 3.6-fold (P<0.01) after 2 months of berberine therapy [16]. A randomized, double-blinded, placebo-controlled, multicenter clinical trial performed by us also revealed that, along with a significant reduction of serum interleukin (IL)-6 levels, berberine treatment significantly improved insulin sensitivity as revealed using hyperinsulinemic euglycemic clamps [15]. However, the primary target of berberine regarding the improvement of insulin sensitivity remains to be elucidated.

Alleviation of inflammation has been identified as an important mechanism in the insulin-sensitizing effects of berberine because of the increasingly evident causative relationship between inflammation and insulin resistance [39]. Pro-inflammatory cytokines, particularly tumor necrosis factor-α (TNF-α), can enhance the serine phosphorylation of insulin receptor substrate-1, a crucial event in the induction of insulin resistance [40]. The anti-inflammatory activities of berberine have been widely reported. Jeong *et al*. [41] reported that berberine significantly suppressed the expression of pro-inflammatory genes, including TNF-α, IL-1β, IL-6, MCP-1, inducible nitric oxide synthase, and cyclooxygenase-2 , in the white adipose tissue of *db/db* mice. Another study utilized a LPS-injured rat model to demonstrate that berberine significantly reduced LPS-induced intestinal damage and decreased serum levels of downstream inflammatory cytokines [42]. In this study, we also observed that berberine co-administration significantly prevented HFD-induced systemic inflammation. Together with previous reports, we suggest that the alleviation of inflammation may serve as an important mechanism in insulin sensitization in berberine-treated rats.

Accumulating evidence indicates that the gut microbiota plays a pivotal role in modulating host immune systems [7]. Structural imbalances of the gut microbiota, particularly reductions in the abundance of gut barrier-protecting bacteria such as *Bifidobacterium* spp. and increases in the abundance of Gram-negative endotoxin-producing bacteria such as *Desulfovibrio* spp., may lead to increases in intestinal permeability and circulating gut-originated antigens, primarily LPS [7], [43]. Upon binding to the complex of CD14 and toll-like receptor 4 on the surface of innate immune cells, LPS can induce systemic inflammation, which eventually impairs insulin sensitivity and induces insulin resistance-related metabolic disorders [7]. Further studies demonstrated that selective increases in the levels of *Bifidobacterium* spp. via the administration of prebiotics completely abolished the metabolic disorders induced by HFD, possibly by alleviating low-grade inflammation and insulin resistance [11]. In the present study, we measured the serum concentration of LBP, a biomarker of circulating LPS [33], to investigate whether there is a possible role of berberine-mediated modulation of the gut microbiota in the alleviation of host inflammation and amelioration of insulin resistance-related metabolic abnormalities. In accordance with the results previously reported by Cani *et al*. [7], HFD induced a significant increase in serum LBP levels in our rats, which was essentially prevented by berberine co-administration, suggesting a potential role of antigens derived from the gut microbiota in this pharmacological process.

To study the detailed structural modulation effects of berberine on the gut microbiota and its possible role in alleviating HFD-induced metabolic deteriorations, we performed a MiWAS based on bar-coded 454 pyrosequencing of the V3 region of 16S rRNA genes and multivariate statistics. Significant reductions in bacterial diversity and the total bacterial population were observed in berberine-treated rats. RDA identified 268 key OTUs that were modulated in response to berberine treatment, most of which were eliminated or inhibited by berberine. Berberine has already been reported to have a wide antibacterial spectrum including some opportunistic pathogens, such as *Staphylococcus*, *Streptococcus*, *Salmonella*, *Klebsiella*, and *Pseudomonas* [44]. The inhibition of a wide range of intestinal bacteria by berberine might result in a decrease of the free antigen load in the host, as confirmed by the decreased serum LBP levels in berberine-treated HFD-fed rats in this study.

Among the 268 key OTUs identified by RDA, approximately one-third were significantly enriched by berberine treatment. Most significantly, key OTUs in the SCFA-producing genera of *Blautia* [45] and *Allobaculum* [46] were enriched by approximately 10-fold compared to their levels in the untreated HFD-fed rats. Determination of fecal SCFA levels by using gas chromatography also indicated that berberine administration significantly increased SCFA concentrations in HFD-fed rats. These results suggest that oral administration of berberine enriches the abundance of SCFA producers to promote colonic fermentation and SCFA production in the intestines of HFD-fed rats. Turnbaugh *et al*. reported that the obese mice with increased Firmucutes and decreased Bacteriodetes in their guts had an elevated colonic fermentation and SCFA production, which might contribute to obesity by increasing the host's capacity for energy harvesting from foods [5]. However, later studies showed that the relationship between the gut microbial composition, energy harvesting capacity and fecal SCFA levels is more complicated. For example, Murphy *et al*. showed that the compositional changes of the major phyla Firmicutes, Bacteroidetes and Actinobacteria were unrelated to markers of energy harvest, and the fecal SCFA levels and fecal energy contents in HFD-fed mice were not correlated [47].

Accumulating reports have focused on the alleviating effects of SCFAs on inflammation and their protective effects on gut barrier function. As an important energy source for intestinal epithelial cells, SCFAs improve gut barrier function by either promoting cell differentiation, facilitating tight junction assembly, or upregulating proglucagon gene expression in intestinal L cells [48], [49]. Indeed, the gut barrier-protecting effects of berberine have been reported in animal models challenged with pro-inflammatory cytokines or LPS [42], [50], the mechanisms of which have been suggested to be the promotion of proglucagon mRNA expression and L cell proliferation in the intestine [27], [28]. Our findings suggest that this gut barrier-protecting function of berberine is mediated by elevated levels of SCFAs produced by selectively enriched SCFA producers in the gut.

Anti-inflammation is another well-characterized function of SCFAs. Increased intake of SCFAs has been reported to be clinically beneficial in the treatment of colitis [51]. Follow-up studies suggested that the G-protein coupled receptor 43, a receptor of SCFAs, mediated the effects of SCFAs in regulating inflammatory responses [52]. Another study by Fukuda *et al*. revealed that SCFAs (namely acetate) produced by certain *Bifidobacterium* strains promoted the defense functions of host epithelial cells and thereby protected the host against lethal infection with enterohemorrhagic *Escherichia coli* O157:H7 [53]. A comparative study in children from Europe and rural Africa also suggested that enrichment of SCFA-producing bacteria such as *Prevotella* and *Xylanibacter* and increased fecal SCFA concentrations in the intestines of rural Africa children help to inhibit opportunistic pathogens such as *Shigella* and *Escherichia* and protect children against inflammation and noninfectious colonic diseases [54]. In this study, significant enrichment of SCFA-producing bacteria and decreased systemic inflammation in berberine-treated HFD-fed rats were also observed. Taken together, the beneficial effects of SCFAs, namely improving gut barrier functions, ameliorating systemic inflammation, or creating a non-permissive environment for pathogens, may mediate the pharmacological effects of berberine against obesity and insulin resistance-related metabolic abnormalities.

Accumulating evidence suggested that diet composition and calorie intake might play an important role in shaping the gut microbiota and modulating host phenotypes [55], [56], [57]. Ravussin *et al*. demonstrated that, apart from effectively decreasing body weight and fat mass, calorie restriction significantly increased the gut microbial diversity and the relative abundance of *Allobaculum* in HFD-fed animals, but not in those of NCD-fed [56]. In the current study, berberine significantly reduced the food intake of HFD-fed rats, which is in accordance with some previous reports [23], [58]. Similar to the calorie-restricted HFD-fed animals, selective enrichment of the genus *Allobaculum* was also observed in berberine-treated HFD-fed rats. However, selective increase of the genus *Blautia* was unique to berberine, and dramatic decrease of the microbial diversity and significant improvement of insulin sensitivity in berberine-treated HFD-fed rats was also observed. This indicates that although the reduction of food intake may contribute in part to the gut microbiota changes or host metabolic phenotype improvements, the direct modulating effects of berberine on gut microbiota may be more critical for the observed effects of berberine on the host health.

Modulation of gut microbiota with diet or drugs has been indicated to improve host metabolic phenotypes [4], [27]; however, whether the response of the gut microbiota to these environmental perturbations happens at the phylum or specific phylotype level remains controversial [59]. Ley *et al.* revealed that obese people had fewer Bacteroidetes and more Firmicutes than lean controls [4]. Upon dietary intervention, the ratio of Firmicutes to Bacteroidetes decreased over time as body weight decreased [4]. However, in another similar human study, no association was observed between obesity and the phylum-level changes of the gut microbiota [59]. In a previous report concerning the effects of berberine on the gut microbiota [27], RT-PCR was used to quantify the proportions of Firmicutes and Bacteroidetes to total bacteria, again indicating no significant association between these phylum-level changes with diet types or obesity in mice, although berberine significantly reduced the proportions of both Firmicutes and Bacteroidetes to total bacteria. Accumulating evidence indicates that variations in the species composition of the gut microbiota were related to human obesity [54], [59]. In a previous report [43], we also revealed that the development of metabolic syndromes was relevant to phylotype-specific changes of the gut microbiota. In the present study, we did not find a significant difference in the ratio of Firmicutes to Bacteroidetes between NCD- and HFD-fed rats. Berberine also displayed no significant influence on the proportions of Firmicutes and Bacteroidetes in rats under both feeding conditions. By using the MiWAS strategy, which combines the high-throughput pyrosequencing of 16S rRNA genes with multivariate statistics, we identified 268 key OTUs responding to berberine treatment from a total of 6720 OTUs observed in the samples. PLS regression modeling performed well in predicting the host phenotypes with the abundance data of those identified key OTUs, suggesting a possible close association between those phylotypes and host health phenotypes. In total, 143 and 87 of the 268 OTUs belong to the phyla Firmicutes and Bacteroidetes, respectively. Contrasting responses of the OTUs in the same phylum or even in the same genus were observed. Hence, the present study suggests that phylotype-level profiling of the variation of the gut microbiota by using the MiWAS strategy will serve as a reliable approach for demonstrating the relationship between the gut microbiota and host metabolic phenotypes under diet or drug perturbations.

In conclusion, our findings suggest that marked modulation of gut microbiota by berberine, namely inhibition of a wide range of intestinal microbes and enrichment of some SCFA producers, helps to alleviate systemic inflammation, at least in part, by reducing the antigen load to the host and elevating SCFA levels in the intestine and contributes to the beneficial effects of berberine against insulin resistance, obesity, diabetes, and other metabolic disorders. Functional metagenomic studies and molecular dissection of the host-microbiome cross-talk are needed to further elucidate the mechanisms of action of berberine [3]. This study also suggests that pharmacological or nutritional modulation of gut microbiota is an effective approach for preventive healthcare.

## Materials and Methods

### Drug and diet

Berberine chloride (BBR) was purchased from Sigma-Aldrich, USA and suspended in 0.5% sodium carboxymethylcellulose (CMC-Na, Sigma-Aldrich) before use. Both the NCD (containing 10% fat by energy) and HFD (containing 60% fat by energy) were obtained from Shanghai Laboratory Animal Center (SLAC), Chinese Academy of Sciences (Shanghai, China).

### Animal experiments

Animal experiments in this study were performed at the animal facilities of SLAC and conducted in strict accordance with the Guide for Care and Use of Laboratory Animals of SLAC, Chinese Academy of Sciences. The protocol was approved by the Institutional Animal Care and Use Committee of SLAC (No. 2011-007). All efforts were made to minimize animal suffering. After 2 weeks of acclimatization, 40 male Wistar rats (8 weeks old, specific pathogen-free grade) were randomly divided into 4 groups of 10 animals per group. Two groups of animals were conventionally raised with NCD, and the other two were fed HFD. In each feeding condition, one group of rats was orally administered 100 mg/kg body weight BBR once daily (NCD+BBR or HFD+BBR group), whereas the other two groups were used as controls and treated with an equal volume of 0.5% CMC-Na (NCD or HFD group).

Animal treatments lasted for 18 weeks, during which the body weight and food intake of each animal were measured once a week. Fresh stool samples were collected in weeks 0, 8, and 18 by using a metabolic cage and immediately stored at −80°C for subsequent analysis.

During the 18^th^ week of the experiment, all animals were subjected to the OGTT and ITT according to previously described methods [60]. Briefly, the OGTT was performed after 12 h of food deprivation, after which 2.0 g/kg body weight glucose was administered orally to the rats. Blood samples were taken from the tail to measure blood glucose levels before and 15, 30, 60, and 120 min after glucose administration by using an *ACCU*-Check glucose meter (Roche Diagnostics, Canada). The ITT was conducted after 6 h of food deprivation, which was followed by intraperitoneal injection of 1.5 U/kg body weight insulin (Humulin®R, Eli Lilly, Egypt). Blood glucose was measured as described for the OGTT.

At the end of the trial, after 12 h of food deprivation, blood was collected from the orbital plexus, and serum was isolated by centrifugation at 3000 rpm at 4°C for 15 min. ELISA kits were used to determine FINS (Mercodia, Sweden), LBP (Cell Sciences, USA) and adiponectin levels (Invitrogen, USA). MCP-1 and leptin levels were simultaneously determined using the Milliplex Map kit (Millipore, USA). All animals were sacrificed by cervical dislocation. Perirenal and epididymal fat pads were excised, weighed, and frozen in liquid nitrogen immediately after sacrifice.

### Fecal DNA extraction and pyrosequencing

Genomic DNA was extracted from fecal samples by bead beating and using a InviMag® Stool DNA kit (Invitek, Germany). Briefly, approximately 0.2 g of thawed feces were added to a 2-ml screw-cap tube containing 1 ml of lysis buffer P of the kit and 0.3 g Zirconia beads (0.1 mm, Biospec Products, Inc., USA). After sufficient homogenization by vortex for approximately 5 min, bead beating was performed for 1 min at full speed. DNA was extracted by following the manufacturer's instructions for bacterial DNA extraction involving proteinase K treatment and subsequent purification using the KingFisher device (Invitek, Germany). The extracted DNA from each sample was used as the template to amplify the V3 region of 16S rRNA genes. PCR amplification, pyrosequencing of the PCR amplicons, and quality control of raw data were performed as described previously [31], [43].

### Bioinformatics and multivariate statistics

All high-quality pyrosequencing sequences were clustered using CD-hit with 99% similarity [61]. The most abundant sequence of each cluster was selected as a representative, aligned against the Greengenes database using the nearest alignment space termination (NAST) algorithm [62], and then imported into the ARB to construct a neighbor-joining tree [63]. The OTU was delineated at 98% similarity level with DOTUR [64]. The most abundant sequence of each OTU was selected as the representative sequence and subjected to RDP classifier for taxonomical assignment with a bootstrap cutoff of 50% [65]. The representative sequences, together with the abundance data (normalized for each sample and log-transformed), were used for further analysis.

The Shannon-Wiener index, Simpson's diversity index, Buzas-Gibson's evenness, and Chao1 and rarefaction estimates were calculated using QIIME [66]. Representative sequences of OTUs were inserted into a pre-established phylogenetic tree of the full-length 16S rRNA gene sequences to generate a phylogenetic tree using ARB [63]. The phylogenetic tree was then used for both weighted and unweighted UniFrac PCoA [67]. The statistical significance of the separation among groups was assessed by MANOVA using the PCA or PCoA scores in MATLAB 2010b (The MathWorks, Inc., USA).

RDA was performed using CANOCO for Windows 4.5 (Microcomputer Power, USA) according to the manufacturer's instructions [68]. Statistical significance was assessed by MCPP with 499 random permutations under the full model.

PLS regression was used to assess the relationship between the gut microbiota structure and host phenotypes. The predictive performance of the PLS model was evaluated by leave-one-out cross-validation, as expressed as the root mean square error of cross-validation and goodness of prediction (Q^2^) [69]. The predictability was also evaluated by the Pearson's correlation coefficient (R) and P-value between predicted and observed host phenotype values. All PLS and correlation methods were performed with MATLAB 2010b.

### Fecal SCFA quantification by using gas chromatography

For fecal SCFA analysis, fecal water was prepared by reconstituting feces in 0.01 M PBS followed by centrifugation (9000×*g*, 5 min at 4°C). The supernatant was acidified with a 1/10 volume of 50% H_2_SO_4_ and extracted with ethyl ether. The concentrations of the SCFAs, including acetic acid, propionic acid, butyric acid, *n*-valeric acid, isobutyric acid, and isovaleric acid, were determined in the organic phase using an Agilent 6890N gas chromatograph equipped with a polar HP-FFAP capillary column (0.25 mm×0.25 mm×30 m) and flame ionization detector (Agilent Technologies, USA). Helium was used as the carrier gas. The initial oven temperature was 140°C, which was maintained for 10 min and then raised to 165°C at 5°C/min, increased to 270°C at 25°C/min, and held at this temperature for 2 min. The detector temperature was 280°C, and the injector temperature was 250°C. Data handling was performed with an Agilent ChemStation (version G2070AA, Agilent Technologies).

### Accession number of sequences

The sequence information from pyrosequencing has been uploaded to the Sequence Read Archive database under the accession number SRP010378.

## Supporting Information

Materials and Methods S1 (DOC)

Figure S1 **Evaluation of the sequencing depth in each sample.** (A–C) Shannon diversity index curves of the samples at weeks 0, 8, and 18. (D–F) Rarefaction curves of the samples at weeks 0, 8, and 18.
(TIF)

Figure S2 **Overall structural changes of the gut microbiota in rats evaluated by PCA and weighted UniFrac analysis.** (A) PCA score plot. Each point represents the mean principal component scores of all rat in a group at one time point, and the error bar represents the standard derivation. (B) Clustering of gut microbiota based on distances between different groups calculated by MANOVA, ^*^ P<0.05. (C) PCoA score plot based on weighted UniFrac metrics. Each point represents the mean principal component scores of all rat in a group at a time point, and the error bar represents the standard derivation. (D) Clustering of the gut microbiota based on distances between different groups calculated by MANOVA, ^*^ P<0.05.
(TIF)

Figure S3 **Richness and diversity of the gut microbiota.** (A) Simpson's index (1-Dominant). (B) Buzas and Gibson's evenness. (C) OTU estimates via rarefaction analysis. (D) Chao1 richness estimates. Calculations were performed after rarefying an equal number of sequence reads for all samples. Values are expressed as means ± standard error. ^*^ P<0.05, significant difference when compared with week 0 data as assessed by ANOVA.
(TIF)

Figure S4 **Quantification of the copies of 16S rRNA genes of total bacteria by real-time PCR.** The median (central thick lines), 25% and 75% quartile ranges (box width), and upper and lower limits (error bar) of each group are shown in the box plot. Differences were analyzed by the Mann-Whitney test. ^*^ P<0.05; ^**^ P<0.01; *^NS^* not significant.
(TIF)

Table S1 Significantly different phyla or genera between groups as revealed by taxon-based comparisons.
(DOC)

Table S2 Taxonomic assignments of 268 key OTUs identified by RDA.
(DOC)

Table S3 Univariate comparisons of the 268 key OTUs identified by RDA.
(DOC)
