# Supplementary material for: Structural Changes of Gut Microbiota during Berberine-Mediated Prevention of Obesity and Insulin Resistance in High-Fat Diet-Fed Rats
Source: PLoS One. 2012 Aug 3;7(8):e42529. doi: 10.1371/journal.pone.0042529 (PMC3411811; doi:10.1371/journal.pone.0042529)
Supplement: Materials and Methods S1 — (DOC) [file pone.0042529.s001.doc]

**Supporting Materials and Methods**

**Real-time PCR for quantifying the copies of 16S rRNA genes of total bacteria**

The total genomic DNA of each sample was subjected to real-time PCR according to a previously described method . Briefly, a plasmid containing a full-length 16S rRNA gene was serially diluted to construct standard curves. PCR was performed in a 25-μl reaction mixture consisting of 12.5 μl iQ SYBR Green SuperMix (Bio-Rad, Richmond, USA), 12.5 pmol of each primer (331F: 5′-TCCT ACGG GAGG CAGC AGT-3′; 797R: 5′-GGAC GGAC TACC AGGG TATC TAAT CCTG TT-3′), and 20 ng of template. The PCR program was initiated at 95°C for 4 min, followed by 40 thermal cycles of 15 s at 95°C, 60 s at 60°C, and 5 s at 80°C for reading the template. Meting curve analysis of PCR products (65–95°C with a heating rate of 0.5°C per/s and a continuous fluorescence measurement) was performed immediately after real-time PCR. Data analysis was performed with Opticon Monitor Software (Version 3.1, MJ Research). Total bacteria levels are expressed as the copies of 16S rRNA genes per gram of wet feces.

**Taxon-based analysis for comparisons of the gut microbiota**

All high-quality pyrosequencing sequences were clustered using CD-hit with 99% similarity . The most abundant sequence of each cluster was selected as a representative and subjected to RDP classifier for taxonomical assignment with a bootstrap cutoff of 50% . The relative abundances of different phyla and genera in each sample were calculated and compared between groups by using the Mann-Whitney test.

**References**

1. Wang T, Cai G, Qiu Y, Fei N, Zhang M, et al. (2011) Structural segregation of gut microbiota between colorectal cancer patients and healthy volunteers. ISME J 6: 320-329.

2. Li W, Godzik A (2006) Cd-hit: a fast program for clustering and comparing large sets of protein or nucleotide sequences. Bioinformatics 22: 1658-1659.

3. Wang Q, Garrity GM, Tiedje JM, Cole JR (2007) Naive Bayesian classifier for rapid assignment of rRNA sequences into the new bacterial taxonomy. Appl Environ Microbiol 73: 5261-5267.
