# Supplementary figures and images for: Structural Changes of Gut Microbiota during Berberine-Mediated Prevention of Obesity and Insulin Resistance in High-Fat Diet-Fed Rats
Source: PLoS One. 2012 Aug 3;7(8):e42529. doi: 10.1371/journal.pone.0042529 (PMC3411811; doi:10.1371/journal.pone.0042529)

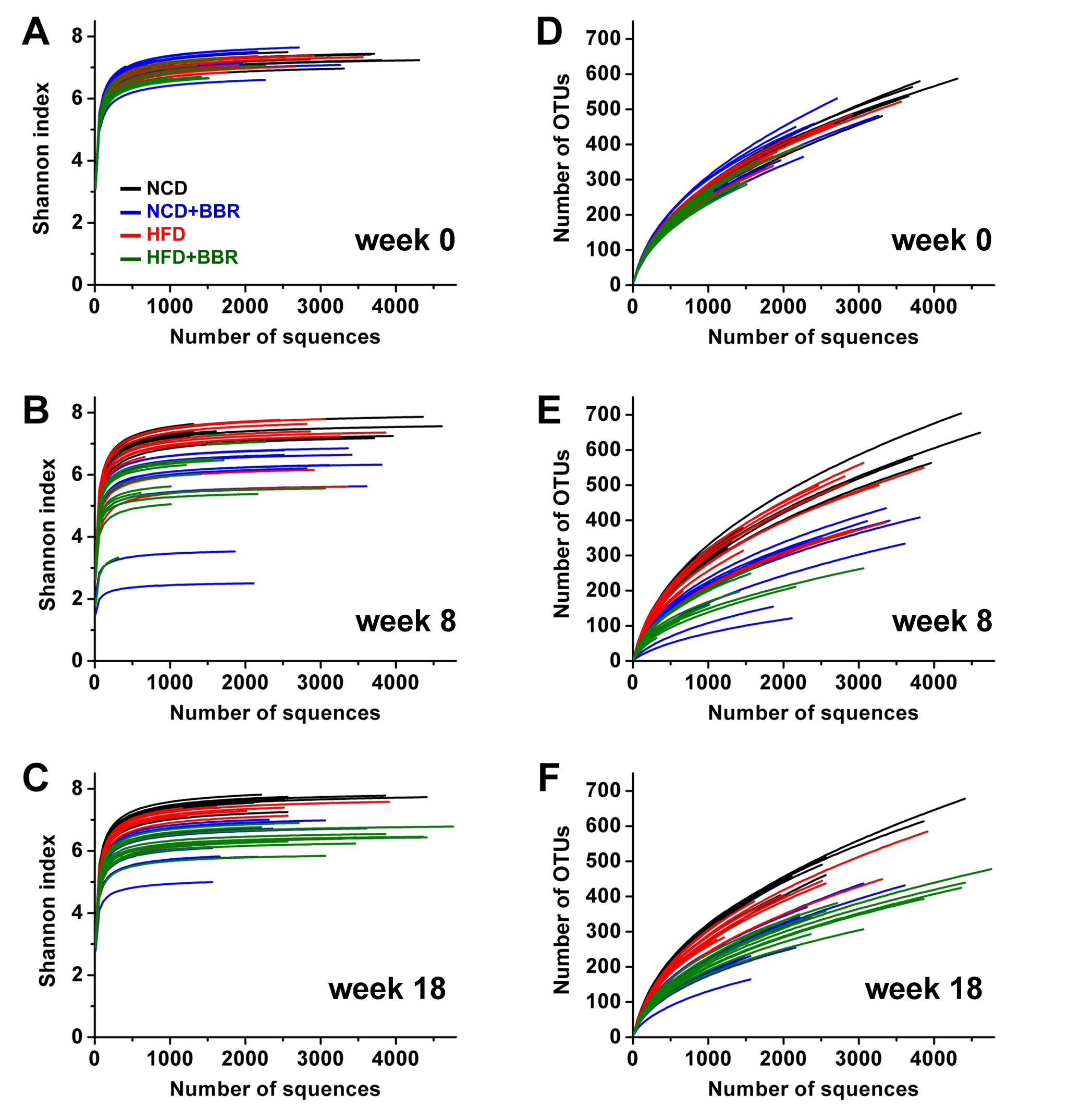

Supplement: Figure S1 — Evaluation of the sequencing depth in each sample. (A–C) Shannon diversity index curves of the samples at weeks 0, 8, and 18. (D–F) Rarefaction curves of the samples at weeks 0, 8, and 18. (TIF) [file pone.0042529.s002.tif]

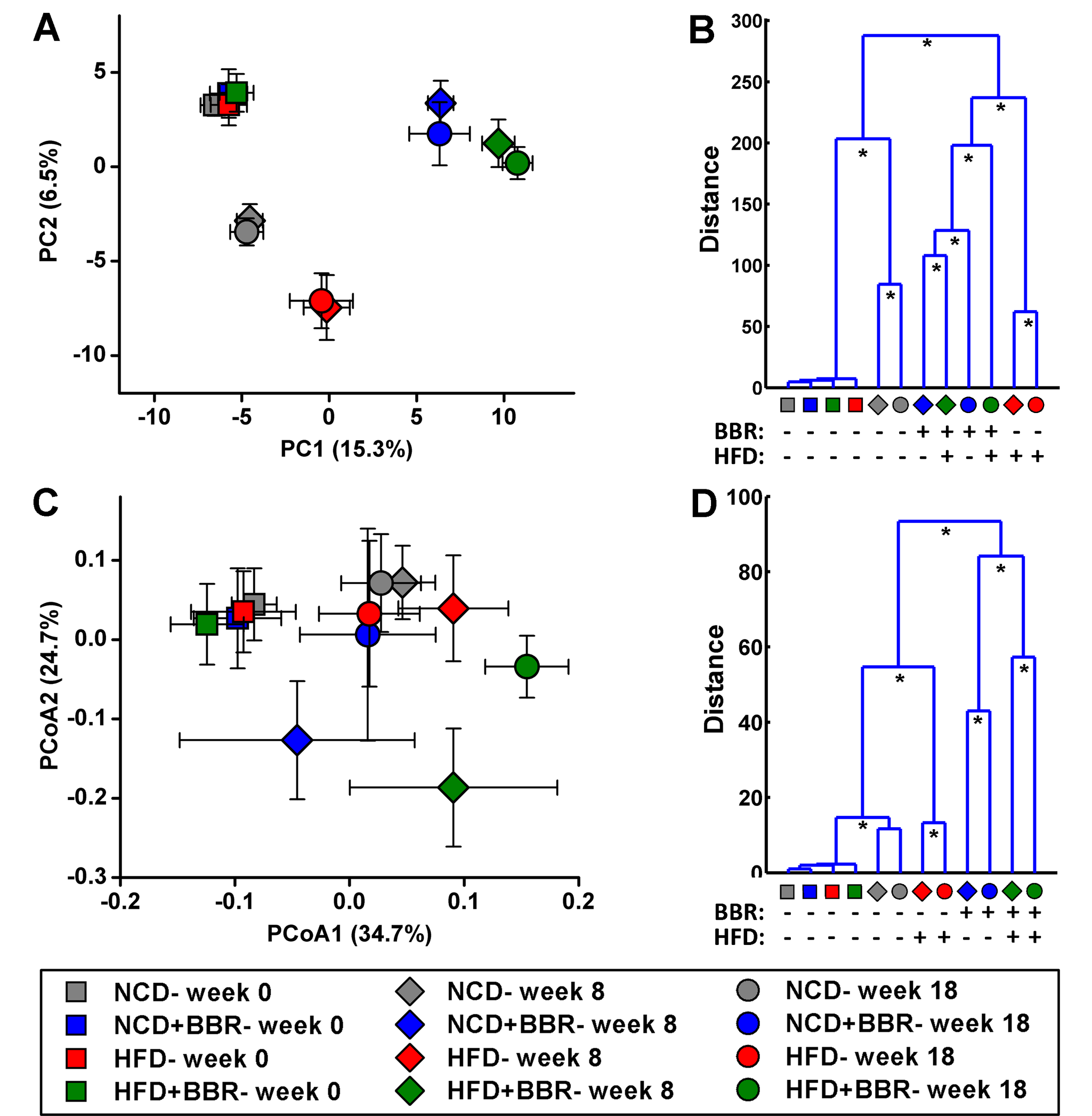

Supplement: Figure S2 — Overall structural changes of the gut microbiota in rats evaluated by PCA and weighted UniFrac analysis. (A) PCA score plot. Each point represents the mean principal component scores of all rat in a group at one time point, and the error bar represents the standard derivation. (B) Clustering of gut microbiota based on distances between different groups calculated by MANOVA, * P<0.05. (C) PCoA score plot based on weighted UniFrac metrics. Each point represents the mean principal component scores of all rat in a group at a time point, and the error bar represents the standard derivation. (D) Clustering of the gut microbiota based on distances between different groups calculated by MANOVA, * P<0.05. (TIF) [file pone.0042529.s003.tif]

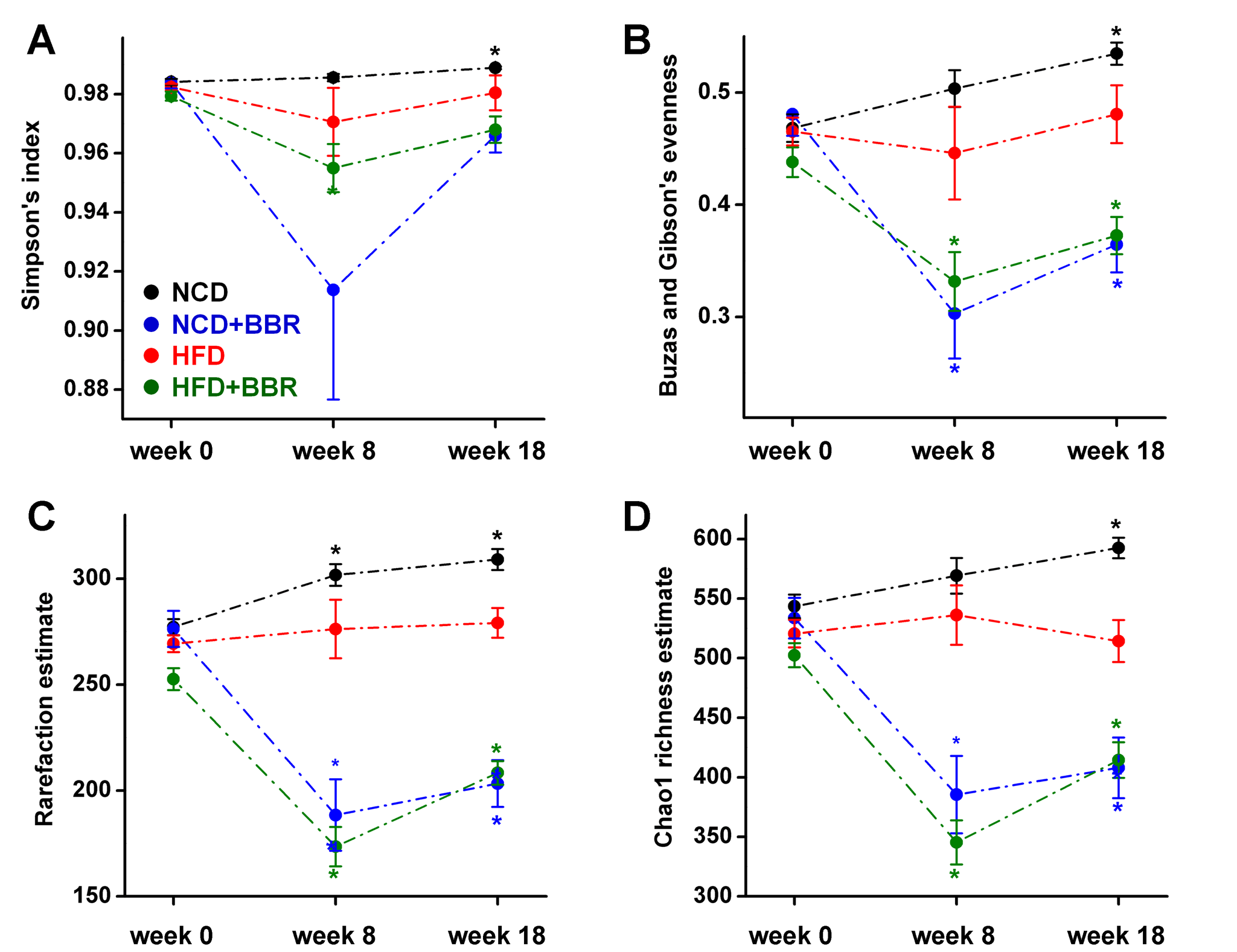

Supplement: Figure S3 — Richness and diversity of the gut microbiota. (A) Simpson's index (1-Dominant). (B) Buzas and Gibson's evenness. (C) OTU estimates via rarefaction analysis. (D) Chao1 richness estimates. Calculations were performed after rarefying an equal number of sequence reads for all samples. Values are expressed as means ± standard error. * P<0.05, significant difference when compared with week 0 data as assessed by ANOVA. (TIF) [file pone.0042529.s004.tif]

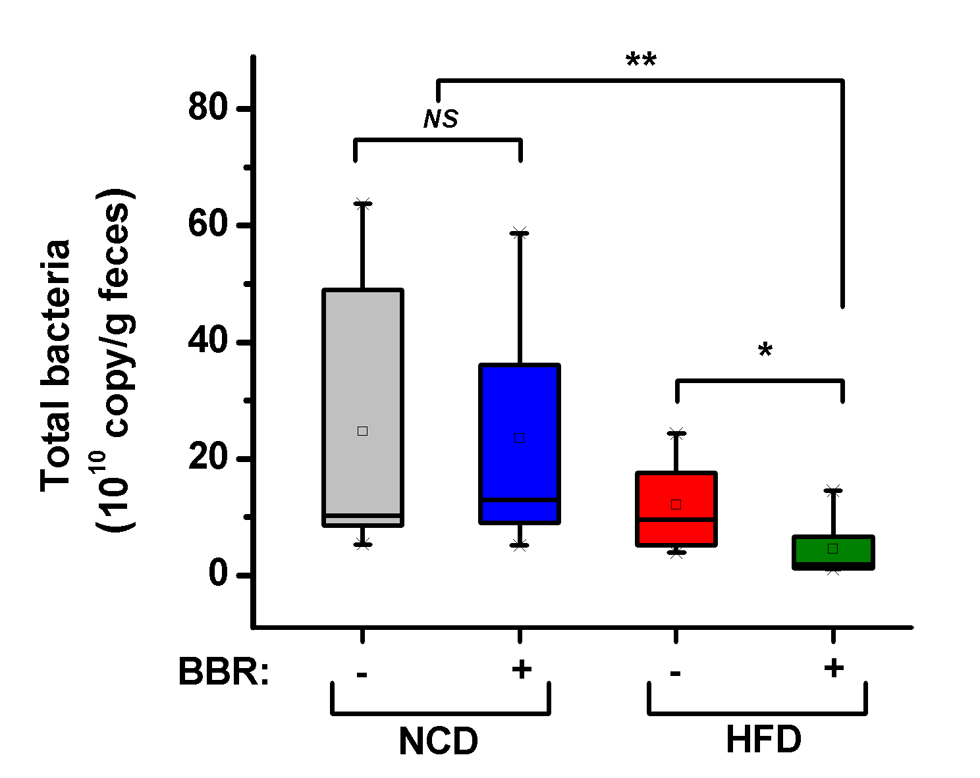

Supplement: Figure S4 — Quantification of the copies of 16S rRNA genes of total bacteria by real-time PCR. The median (central thick lines), 25% and 75% quartile ranges (box width), and upper and lower limits (error bar) of each group are shown in the box plot. Differences were analyzed by the Mann-Whitney test. * P<0.05; ** P<0.01; NS not significant. (TIF) [file pone.0042529.s005.tif]
