# Supplementary material for: Structural Changes of Gut Microbiota during Berberine-Mediated Prevention of Obesity and Insulin Resistance in High-Fat Diet-Fed Rats
Source: PLoS One. 2012 Aug 3;7(8):e42529. doi: 10.1371/journal.pone.0042529 (PMC3411811; doi:10.1371/journal.pone.0042529)
Supplement: Table S1 — Significantly different phyla or genera between groups as revealed by taxon-based comparisons. (DOC) [file pone.0042529.s006.doc]

**Table S1** Significantly different phyla or genera between groups as revealed by taxon-based comparisons

|  | **P values calculated by Mann-Whitney test** | | | | | | **Median (first quart, third quart) %** | | | |
| --- | --- | --- | --- | --- | --- | --- | --- | --- | --- | --- |
| **Taxa** | **NCD**  **vs.**  **NCD+BBR** | **NCD**  **vs.**  **HFD** | **NCD**  **vs.**  **HFD+BBR** | **NCD+BBR**  **vs.**  **HFD** | **NCD+BBR**  **vs.**  **HFD+BBR** | **HFD**  **vs.**  **HFD+BBR** | **NCD** | **NCD+BBR** | **HFD** | **HFD+BBR** |
| ***Phylum level*** | | | | | | | | | | |
| *Actinobacteria* | 0.0140 | 0.0312 | 0.7337 | 0.0002 | 0.0036 | 0.0173 | 0.51(0.2, 1.46) | 0.08(0.05, 0.1) | 2.54(0.86, 5.64) | 0.56(0.31, 1) |
| *Bacteroidetes* | 0.1405 | 0.6776 | 0.2123 | 0.3075 | 0.0073 | 0.1405 | 27.63(22.76, 32.98) | 32.29(26.44, 50.29) | 32.52(18.01, 39.41) | 21.3(18.77, 27.92) |
| *Firmicutes* | 0.9097 | 1.0000 | 0.0173 | 0.6776 | 0.0452 | 0.0539 | 58.28(54.38, 62.38) | 61.5(44.18, 67.37) | 58.02(48.55, 70.09) | 68.34(65.23, 75.6) |
| *Fusobacteria* | 0.1012 | 0.6082 | 0.2917 | 0.5036 | 0.0056 | 0.0394 | 0(0, 0.05) | 0(0, 0) | 0(0, 0) | 0.04(0.03, 0.1) |
| *Synergistetes* | 0.3840 | 0.0002 | 0.0001 | 0.0091 | 0.0022 | 0.1681 | 0.06(0.04, 0.15) | 0.06(0.01, 0.08) | 0(0, 0) | 0(0, 0) |
| *TM7* | 0.0022 | 0.0724 | 0.0049 | 0.0149 | 0.3681 | 0.0623 | 0.13(0.03, 0.38) | 0(0, 0) | 0.01(0, 0.07) | 0(0, 0) |
| *Tenericutes* | 0.0008 | 0.0110 | 0.0008 | 0.1681 | 1.0000 | 0.1681 | 0.27(0.07, 0.53) | 0(0, 0) | 0(0, 0) | 0(0, 0) |
| *Verrucomicrobia* | 0.3681 | 0.0002 | 0.3681 | 0.0009 | 1.0000 | 0.0007 | 0(0, 0) | 0(0, 0) | 0.21(0.04, 0.32) | 0(0, 0) |
| *Bacteroidetes/Firmicutes* | 0.3847 | 0.7337 | 0.0757 | 0.4274 | 0.0173 | 0.0890 | 0.47(0.37, 0.65) | 0.53(0.39, 1.13) | 0.58(0.26, 0.87) | 0.32(0.27, 0.41) |
| ***Genus level*** | | | | | | | | | | |
| *Acetanaerobacterium* | 0.0149 | 0.3059 | 0.4318 | 0.0779 | 0.0350 | 0.7562 | 0.02(0, 0.2) | 0(0, 0) | 0(0, 0.02) | 0(0, 0.04) |
| *Acetivibrio* | 0.0779 | 0.7281 | 0.1249 | 0.1681 | 0.0022 | 0.1476 | 0(0, 0.02) | 0(0, 0) | 0(0, 0) | 0.04(0.01, 0.11) |
| *Akkermansia* | 0.3681 | 0.0002 | 0.3681 | 0.0009 | 1.0000 | 0.0007 | 0(0, 0) | 0(0, 0) | 0.21(0.04, 0.32) | 0(0, 0) |
| *Alistipes* | 0.0890 | 0.2116 | 0.3447 | 0.0373 | 0.5708 | 0.0752 | 0.31(0.21, 0.55) | 0.52(0.44, 0.67) | 0.11(0.01, 0.5) | 0.49(0.34, 0.66) |
| *Allobaculum* | 0.4495 | 0.0283 | 0.0002 | 0.2897 | 0.0003 | 0.0004 | 0.25(0.14, 0.46) | 0.61(0.1, 1.04) | 1.6(0.52, 2.42) | 9.07(6.84, 10.15) |
| *Anaerobacter* | 0.3744 | 0.0187 | 0.1971 | 0.0069 | 0.0299 | 0.1986 | 0.06(0.01, 0.15) | 0.04(0, 0.06) | 0.5(0.17, 0.86) | 0.12(0.09, 0.52) |
| *Anaerofilum* | 0.1975 | 0.5005 | 0.0044 | 0.5522 | 0.4710 | 0.0866 | 0.04(0, 0.05) | 0.09(0, 0.27) | 0.04(0, 0.11) | 0.15(0.07, 0.2) |
| *Anaeroplasma* | 0.0008 | 0.0110 | 0.0008 | 0.1681 | 1.0000 | 0.1681 | 0.27(0.07, 0.53) | 0(0, 0) | 0(0, 0) | 0(0, 0) |
| *Anaerosporobacter* | 0.0107 | 0.0168 | 0.6629 | 0.6776 | 0.0386 | 0.0362 | 0.08(0, 0.21) | 0.57(0.23, 1.42) | 0.71(0.12, 1.04) | 0.01(0, 0.11) |
| *Anaerotruncus* | 0.0822 | 1.0000 | 0.2106 | 0.1598 | 0.0185 | 0.3410 | 0.05(0.04, 0.05) | 0.01(0, 0.04) | 0.04(0.01, 0.1) | 0.09(0.05, 0.13) |
| *Asaccharobacter* | 0.5036 | 0.4283 | 0.0752 | 0.1236 | 0.0049 | 0.2193 | 0(0, 0) | 0(0, 0) | 0(0, 0.05) | 0.04(0.01, 0.06) |
| *Atopobium* | 0.1681 | 0.1008 | 1.0000 | 0.0149 | 0.1681 | 0.1206 | 0(0, 0) | 0(0, 0) | 0.01(0, 0.08) | 0(0, 0) |
| *Bacteroides* | 0.0376 | 0.0452 | 0.0003 | 0.9698 | 0.0890 | 0.0539 | 0.98(0.54, 1.49) | 1.9(1.33, 4.76) | 3.46(1.28, 4.38) | 5.29(3.57, 5.67) |
| *Barnesiella* | 0.0008 | 0.7337 | 0.0312 | 0.0113 | 0.3075 | 0.1212 | 4.25(3.35, 5.73) | 8.54(6.89, 17.98) | 4.5(2.98, 7.5) | 8.18(5.59, 8.66) |
| *Bifidobacterium* | 0.0049 | 0.0310 | 0.1679 | 0.0001 | 0.0105 | 0.0006 | 0.13(0.02, 1) | 0(0, 0) | 1.96(0.65, 4.94) | 0.04(0.01, 0.1) |
| *Blautia* | 0.0025 | 0.0028 | 0.0002 | 0.1212 | 0.0113 | 0.0036 | 0.22(0.07, 0.24) | 1.7(1.45, 3.38) | 0.76(0.55, 1.96) | 7.35(3.55, 14.42) |
| *Butyricicoccus* | 0.0043 | 0.2640 | 0.0043 | 0.0023 | 0.8798 | 0.0030 | 0.04(0, 0.05) | 0.23(0.13, 0.29) | 0(0, 0.03) | 0.19(0.11, 0.51) |
| *Butyricimonas* | 0.5708 | 0.5706 | 0.0022 | 0.2723 | 0.9097 | 0.0032 | 0.5(0.28, 0.68) | 1.77(0.18, 3.89) | 0.32(0.25, 0.57) | 1.61(0.96, 2.25) |
| *Butyrivibrio* | 0.0660 | 0.5400 | 0.6065 | 0.1986 | 0.0016 | 0.0034 | 0.02(0, 0.28) | 0.22(0.1, 1.67) | 0.15(0.08, 0.23) | 0.02(0, 0.05) |
| *Coprobacillus* | 0.3305 | 0.8521 | 0.2257 | 0.3305 | 0.0265 | 0.3743 | 0(0, 0.04) | 0(0, 0) | 0(0, 0.07) | 0.03(0.01, 0.07) |
| *Desulfovibrio* | 0.7593 | 0.0735 | 0.0017 | 0.0435 | 0.0008 | 0.4725 | 0.12(0.02, 0.19) | 0.06(0.01, 0.22) | 0.75(0.13, 1.16) | 0.62(0.41, 1.48) |
| *Dorea* | 0.4283 | 0.9660 | 0.0009 | 0.4846 | 0.0005 | 0.0052 | 0(0, 0.07) | 0(0, 0) | 0(0, 0.04) | 0.28(0.19, 0.37) |
| *Enterorhabdus* | 0.0779 | 0.2343 | 0.0237 | 0.3681 | 0.0022 | 0.0049 | 0(0, 0.03) | 0(0, 0) | 0(0, 0) | 0.2(0.01, 0.34) |
| *Ethanoligenens* | 0.3903 | 0.2378 | 0.0255 | 0.0822 | 0.0086 | 0.4725 | 0.05(0.01, 0.12) | 0.02(0, 0.04) | 0.1(0.04, 0.34) | 0.21(0.09, 0.33) |
| *Eubacterium* | 0.0008 | 0.0019 | 0.0260 | 0.3681 | 0.0350 | 0.1497 | 0.09(0.03, 0.14) | 0(0, 0) | 0(0, 0) | 0(0, 0.02) |
| *Fastidiosipila* | 0.0002 | 0.0036 | 0.0376 | 0.1857 | 0.0008 | 0.0312 | 0.72(0.55, 1) | 0.14(0.06, 0.17) | 0.17(0.13, 0.27) | 0.43(0.28, 0.55) |
| *Fusobacterium* | 0.2343 | 0.8038 | 0.0884 | 0.5036 | 0.0056 | 0.0323 | 0(0, 0.04) | 0(0, 0) | 0(0, 0) | 0.04(0.03, 0.1) |
| *Gordonibacter* | 0.0149 | 0.0934 | 0.0149 | 0.3681 | 1.0000 | 0.3681 | 0.01(0, 0.04) | 0(0, 0) | 0(0, 0) | 0(0, 0) |
| *Hallella* | 0.0014 | 1.0000 | 0.0371 | 0.0025 | 0.0238 | 0.0460 | 0(0, 0) | 0.11(0.06, 0.17) | 0(0, 0) | 0.03(0, 0.04) |
| *Helicobacter* | 0.2730 | 0.0211 | 0.0257 | 0.0004 | 0.0017 | 0.7913 | 0.78(0.67, 2.67) | 1.9(1.34, 3.55) | 0.41(0.2, 0.57) | 0.45(0.3, 0.57) |
| *Holdemania* | 0.5036 | 0.3681 | 0.0014 | 0.1681 | 0.0138 | 0.0008 | 0(0, 0) | 0(0, 0) | 0(0, 0) | 0.1(0.03, 0.12) |
| *Lactobacillus* | 0.4274 | 0.0257 | 0.0757 | 0.0757 | 0.2730 | 0.3075 | 3.22(1.67, 6.31) | 3.08(1.06, 3.97) | 0.81(0.35, 1.22) | 1.33(0.56, 1.81) |
| *Lebetimonas* | 0.3870 | 0.0154 | 0.0292 | 0.0082 | 0.0082 | 0.6458 | 0(0, 0.02) | 0(0, 0) | 0.1(0.01, 0.24) | 0.08(0.01, 0.15) |
| *Marvinbryantia* | 0.3447 | 0.0890 | 0.0036 | 0.6232 | 0.0112 | 0.0017 | 0.58(0.38, 0.8) | 0.77(0.58, 1.4) | 1.11(0.51, 1.67) | 0.09(0.05, 0.26) |
| *Moryella* | 0.0452 | 0.0058 | 0.3447 | 0.1620 | 0.0211 | 0.0013 | 0.22(0.14, 0.38) | 2.15(0.49, 3.37) | 0.61(0.51, 0.76) | 0.17(0.05, 0.29) |
| *Oribacterium* | 0.2302 | 0.1588 | 0.2415 | 0.0100 | 0.8942 | 0.0061 | 0.03(0, 0.08) | 0(0, 0.03) | 0.06(0.04, 0.15) | 0(0, 0.02) |
| *Oscillibacter* | 0.0036 | 0.3075 | 0.7913 | 0.0028 | 0.0036 | 0.9097 | 7.79(7.44, 8.71) | 3.66(2.49, 5.75) | 11.42(6.77, 13.94) | 7.84(6.84, 11.45) |
| *Papillibacter* | 0.0350 | 0.0350 | 0.0350 | 1.0000 | 1.0000 | 1.0000 | 0(0, 0.03) | 0(0, 0) | 0(0, 0) | 0(0, 0) |
| *Parabacteroides* | 0.0752 | 0.0373 | 0.2116 | 0.8798 | 0.4055 | 0.1735 | 0.11(0.04, 0.23) | 0.45(0.08, 0.58) | 0.48(0.24, 0.68) | 0.26(0.05, 0.38) |
| *Parasporobacterium* | 0.0433 | 0.1012 | 0.0185 | 0.0049 | 0.8199 | 0.0005 | 0(0, 0.05) | 0.1(0.02, 0.33) | 0(0, 0) | 0.11(0.05, 0.15) |
| *Parasutterella* | 0.0123 | 0.3440 | 0.5452 | 0.0133 | 0.0152 | 0.9698 | 0.19(0.1, 0.27) | 0.05(0.01, 0.09) | 0.25(0.17, 0.4) | 0.21(0.1, 0.41) |
| *Parvimonas* | 0.2006 | 0.3517 | 0.0079 | 0.8361 | 0.2505 | 0.1530 | 0(0, 0.02) | 0.02(0, 0.06) | 0(0, 0.08) | 0.06(0.03, 0.12) |
| *Phascolarctobacterium* | 0.2440 | 0.6704 | 0.0001 | 0.1799 | 0.0011 | 0.0001 | 0(0, 0) | 0(0, 0.11) | 0(0, 0) | 1.57(0.91, 2.52) |
| *Prevotella* | 0.0004 | 0.6776 | 0.4274 | 0.0058 | 0.0002 | 0.1212 | 0.77(0.66, 1.17) | 5.07(2.74, 15.06) | 1.21(0.6, 1.38) | 0.69(0.15, 0.92) |
| *Roseburia* | 0.6230 | 0.0058 | 0.0640 | 0.1040 | 0.3845 | 0.3847 | 0.36(0.32, 0.61) | 0.62(0.28, 1.2) | 1.31(0.99, 1.88) | 0.99(0.62, 1.45) |
| *Rothia* | 0.0779 | 0.0350 | 0.0779 | 0.5642 | 1.0000 | 0.5642 | 0(0, 0) | 0(0, 0.03) | 0(0, 0.09) | 0(0, 0.02) |
| *Ruminococcus* | 0.0006 | 0.0003 | 0.0015 | 0.4212 | 0.0557 | 0.3964 | 1.65(0.86, 2.25) | 0.25(0.08, 0.37) | 0.07(0, 0.27) | 0(0, 0.1) |
| *Sedimentibacter* | 0.9660 | 0.1236 | 0.1910 | 0.1012 | 0.1910 | 0.0049 | 0(0, 0.04) | 0(0, 0.06) | 0(0, 0) | 0.05(0.01, 0.14) |
| *Sporacetigenium* | 0.9097 | 0.0002 | 0.0028 | 0.0113 | 0.1405 | 0.0890 | 0.34(0.25, 0.42) | 0.3(0.08, 1.59) | 2.66(1.09, 4.63) | 1.15(0.66, 2.27) |
| *Sporobacter* | 0.5349 | 0.0403 | 0.1206 | 0.1236 | 0.4846 | 0.5036 | 0.02(0, 0.08) | 0(0, 0.04) | 0(0, 0) | 0(0, 0) |
| *Streptococcus* | 0.1681 | 0.3278 | 0.6548 | 0.0350 | 0.0779 | 0.5642 | 0(0, 0) | 0(0, 0) | 0(0, 0.08) | 0(0, 0.03) |
| *TM7_genera_*  *incertae_sedis* | 0.0022 | 0.0724 | 0.0049 | 0.0149 | 0.3681 | 0.0623 | 0.13(0.03, 0.38) | 0(0, 0) | 0.01(0, 0.07) | 0(0, 0) |
| *Turicibacter* | 0.8501 | 0.0376 | 0.0006 | 0.0885 | 0.0013 | 0.2730 | 0.11(0.07, 0.18) | 0.14(0.06, 0.23) | 0.37(0.16, 0.87) | 0.7(0.49, 0.95) |
| *Wolinella* | 0.0072 | 0.6704 | 0.0460 | 0.0032 | 0.0848 | 0.0803 | 0(0, 0) | 0.09(0.05, 0.12) | 0(0, 0) | 0.02(0, 0.07) |
