# Supplementary material for: Structural Changes of Gut Microbiota during Berberine-Mediated Prevention of Obesity and Insulin Resistance in High-Fat Diet-Fed Rats
Source: PLoS One. 2012 Aug 3;7(8):e42529. doi: 10.1371/journal.pone.0042529 (PMC3411811; doi:10.1371/journal.pone.0042529)
Supplement: Table S2 — Taxonomic assignments of 268 key OTUs identified by RDA. (DOC) [file pone.0042529.s007.doc]

**Table S2** Taxonomic assignments of 268 key OTUs identified by RDA

| **OTU name** | **Domain** | **Phylum** | **Class** | **Order** | **Family** | **Genus** |
| --- | --- | --- | --- | --- | --- | --- |
| U00436195 | Bacteria | *Proteobacteria* | *Alphaproteobacteria* |  |  |  |
| U00101416 | Bacteria | *Proteobacteria* | *Epsilonproteobacteria* | *Campylobacterales* | *Helicobacteraceae* | *Helicobacter* |
| U00000407 | Bacteria | *Proteobacteria* | *Epsilonproteobacteria* | *Campylobacterales* | *Helicobacteraceae* | *Helicobacter* |
| U00449939 | Bacteria | *Proteobacteria* | *Epsilonproteobacteria* | *Campylobacterales* | *Helicobacteraceae* | *Helicobacter* |
| U01131573 | Bacteria | *Proteobacteria* | *Epsilonproteobacteria* | *Campylobacterales* | *Helicobacteraceae* | *Helicobacter* |
| U00442991 | Bacteria | *Proteobacteria* | *Deltaproteobacteria* | *Desulfovibrionales* | *Desulfovibrionaceae* | *Lawsonia* |
| U01131468 | Bacteria | *Bacteroidetes* | *Bacteroidia* | *Bacteroidales* | *Bacteroidaceae* | *Bacteroides* |
| U00000076 | Bacteria | *Bacteroidetes* | *Bacteroidia* | *Bacteroidales* | *Bacteroidaceae* | *Bacteroides* |
| U00000261 | Bacteria | *Bacteroidetes* | *Bacteroidia* | *Bacteroidales* | *Bacteroidaceae* | *Bacteroides* |
| U00277049 | Bacteria | *Bacteroidetes* | *Bacteroidia* | *Bacteroidales* | *Bacteroidaceae* | *Bacteroides* |
| U01140154 | Bacteria | *Bacteroidetes* | *Bacteroidia* | *Bacteroidales* | *Prevotellaceae* | *Prevotella* |
| U00797917 | Bacteria | *Bacteroidetes* | *Bacteroidia* | *Bacteroidales* | *Prevotellaceae* | *Prevotella* |
| U00800966 | Bacteria | *Bacteroidetes* | *Bacteroidia* | *Bacteroidales* | *Prevotellaceae* | *Prevotella* |
| U01131982 | Bacteria | *Bacteroidetes* | *Bacteroidia* | *Bacteroidales* | *Prevotellaceae* | *Prevotella* |
| U00797839 | Bacteria | *Bacteroidetes* | *Bacteroidia* | *Bacteroidales* | *Prevotellaceae* | *Prevotella* |
| U00003981 | Bacteria | *Bacteroidetes* | *Bacteroidia* | *Bacteroidales* | *Prevotellaceae* |  |
| U00001404 | Bacteria | *Bacteroidetes* | *Bacteroidia* | *Bacteroidales* |  |  |
| U00000052 | Bacteria | *Bacteroidetes* | *Bacteroidia* | *Bacteroidales* |  |  |
| U00000964 | Bacteria | *Bacteroidetes* | *Bacteroidia* | *Bacteroidales* |  |  |
| U00040690 | Bacteria | *Bacteroidetes* | *Bacteroidia* | *Bacteroidales* |  |  |
| U00823991 | Bacteria | *Bacteroidetes* | *Bacteroidia* | *Bacteroidales* |  |  |
| U00000804 | Bacteria | *Bacteroidetes* | *Bacteroidia* | *Bacteroidales* | *Porphyromonadaceae* | *Butyricimonas* |
| U01155915 | Bacteria | *Bacteroidetes* | *Bacteroidia* | *Bacteroidales* | *Porphyromonadaceae* |  |
| U00459850 | Bacteria | *Bacteroidetes* | *Bacteroidia* | *Bacteroidales* | *Porphyromonadaceae* |  |
| U00798098 | Bacteria | *Bacteroidetes* | *Bacteroidia* | *Bacteroidales* | *Porphyromonadaceae* |  |
| U00009170 | Bacteria | *Bacteroidetes* | *Bacteroidia* | *Bacteroidales* | *Porphyromonadaceae* |  |
| U01102153 | Bacteria | *Bacteroidetes* | *Bacteroidia* | *Bacteroidales* | *Porphyromonadaceae* |  |
| U00006230 | Bacteria | *Bacteroidetes* | *Bacteroidia* | *Bacteroidales* | *Porphyromonadaceae* |  |
| U00465344 | Bacteria | *Bacteroidetes* | *Bacteroidia* | *Bacteroidales* | *Porphyromonadaceae* |  |
| U00001202 | Bacteria | *Bacteroidetes* | *Bacteroidia* | *Bacteroidales* | *Porphyromonadaceae* |  |
| U00001384 | Bacteria | *Bacteroidetes* | *Bacteroidia* | *Bacteroidales* | *Porphyromonadaceae* | *Barnesiella* |
| U00802799 | Bacteria | *Bacteroidetes* | *Bacteroidia* | *Bacteroidales* | *Porphyromonadaceae* | *Barnesiella* |
| U00808453 | Bacteria | *Bacteroidetes* | *Bacteroidia* | *Bacteroidales* | *Porphyromonadaceae* | *Barnesiella* |
| U01135802 | Bacteria | *Bacteroidetes* | *Bacteroidia* | *Bacteroidales* | *Porphyromonadaceae* | *Barnesiella* |
| U01199632 | Bacteria | *Bacteroidetes* | *Bacteroidia* | *Bacteroidales* | *Porphyromonadaceae* | *Barnesiella* |
| U01136954 | Bacteria | *Bacteroidetes* | *Bacteroidia* | *Bacteroidales* | *Porphyromonadaceae* | *Barnesiella* |
| U00026711 | Bacteria | *Bacteroidetes* | *Bacteroidia* | *Bacteroidales* | *Porphyromonadaceae* |  |
| U00000650 | Bacteria | *Bacteroidetes* | *Bacteroidia* | *Bacteroidales* |  |  |
| U00000839 | Bacteria | *Bacteroidetes* |  |  |  |  |
| U01157930 | Bacteria | *Bacteroidetes* | *Bacteroidia* | *Bacteroidales* | *Porphyromonadaceae* |  |
| U00798420 | Bacteria | *Bacteroidetes* | *Bacteroidia* | *Bacteroidales* | *Porphyromonadaceae* |  |
| U00001933 | Bacteria | *Bacteroidetes* | *Bacteroidia* | *Bacteroidales* | *Porphyromonadaceae* | *Barnesiella* |
| U00001491 | Bacteria | *Bacteroidetes* | *Bacteroidia* | *Bacteroidales* | *Porphyromonadaceae* |  |
| U00006476 | Bacteria | *Bacteroidetes* | *Bacteroidia* | *Bacteroidales* | *Porphyromonadaceae* |  |
| U01156163 | Bacteria | *Bacteroidetes* | *Bacteroidia* | *Bacteroidales* | *Porphyromonadaceae* | *Butyricimonas* |
| U00001502 | Bacteria | *Bacteroidetes* |  |  |  |  |
| U00001504 | Bacteria | *Bacteroidetes* | *Bacteroidia* | *Bacteroidales* | *Porphyromonadaceae* | *Barnesiella* |
| U00000609 | Bacteria | *Bacteroidetes* | *Bacteroidia* | *Bacteroidales* | *Porphyromonadaceae* |  |
| U00436427 | Bacteria | *Bacteroidetes* | *Bacteroidia* | *Bacteroidales* | *Porphyromonadaceae* |  |
| U00009261 | Bacteria | *Bacteroidetes* | *Bacteroidia* | *Bacteroidales* | *Porphyromonadaceae* |  |
| U01136992 | Bacteria | *Bacteroidetes* | *Bacteroidia* | *Bacteroidales* | *Porphyromonadaceae* |  |
| U01161731 | Bacteria | *Bacteroidetes* | *Bacteroidia* | *Bacteroidales* | *Porphyromonadaceae* | *Barnesiella* |
| U00804243 | Bacteria | *Bacteroidetes* | *Bacteroidia* | *Bacteroidales* | *Porphyromonadaceae* | *Barnesiella* |
| U00832647 | Bacteria | *Bacteroidetes* | *Bacteroidia* | *Bacteroidales* | *Porphyromonadaceae* |  |
| U00000497 | Bacteria | *Bacteroidetes* | *Bacteroidia* | *Bacteroidales* | *Porphyromonadaceae* | *Barnesiella* |
| U00013412 | Bacteria | *Bacteroidetes* | *Bacteroidia* | *Bacteroidales* | *Porphyromonadaceae* | *Butyricimonas* |
| U00000886 | Bacteria | *Bacteroidetes* | *Bacteroidia* | *Bacteroidales* | *Porphyromonadaceae* |  |
| U01143292 | Bacteria | *Bacteroidetes* | *Bacteroidia* | *Bacteroidales* | *Porphyromonadaceae* | *Barnesiella* |
| U00807079 | Bacteria | *Bacteroidetes* | *Bacteroidia* | *Bacteroidales* | *Porphyromonadaceae* | *Barnesiella* |
| U00803188 | Bacteria | *Bacteroidetes* | *Bacteroidia* | *Bacteroidales* | *Porphyromonadaceae* | *Barnesiella* |
| U00008782 | Bacteria | *Bacteroidetes* | *Bacteroidia* | *Bacteroidales* | *Porphyromonadaceae* |  |
| U00005346 | Bacteria | *Bacteroidetes* | *Bacteroidia* | *Bacteroidales* | *Porphyromonadaceae* |  |
| U00004086 | Bacteria | *Bacteroidetes* | *Bacteroidia* | *Bacteroidales* | *Porphyromonadaceae* | *Barnesiella* |
| U00001406 | Bacteria | *Bacteroidetes* | *Bacteroidia* | *Bacteroidales* | *Porphyromonadaceae* |  |
| U00030880 | Bacteria | *Bacteroidetes* | *Bacteroidia* | *Bacteroidales* | *Porphyromonadaceae* | *Barnesiella* |
| U01130438 | Bacteria | *Bacteroidetes* | *Bacteroidia* | *Bacteroidales* | *Porphyromonadaceae* |  |
| U01131459 | Bacteria | *Bacteroidetes* | *Bacteroidia* | *Bacteroidales* | *Porphyromonadaceae* | *Barnesiella* |
| U00436448 | Bacteria | *Bacteroidetes* | *Bacteroidia* | *Bacteroidales* | *Porphyromonadaceae* |  |
| U00474862 | Bacteria | *Bacteroidetes* | *Bacteroidia* | *Bacteroidales* | *Porphyromonadaceae* |  |
| U00450938 | Bacteria | *Bacteroidetes* | *Bacteroidia* | *Bacteroidales* | *Porphyromonadaceae* | *Barnesiella* |
| U00009763 | Bacteria | *Bacteroidetes* | *Bacteroidia* | *Bacteroidales* | *Porphyromonadaceae* | *Barnesiella* |
| U01155495 | Bacteria | *Bacteroidetes* | *Bacteroidia* | *Bacteroidales* | *Porphyromonadaceae* |  |
| U00798694 | Bacteria | *Bacteroidetes* | *Bacteroidia* | *Bacteroidales* | *Porphyromonadaceae* |  |
| U00001388 | Bacteria | *Bacteroidetes* | *Bacteroidia* | *Bacteroidales* | *Porphyromonadaceae* | *Barnesiella* |
| U01185184 | Bacteria | *Bacteroidetes* | *Bacteroidia* | *Bacteroidales* | *Porphyromonadaceae* | *Barnesiella* |
| U00802360 | Bacteria | *Bacteroidetes* | *Bacteroidia* | *Bacteroidales* | *Porphyromonadaceae* |  |
| U01162782 | Bacteria | *Bacteroidetes* | *Bacteroidia* | *Bacteroidales* | *Porphyromonadaceae* |  |
| U00002709 | Bacteria | *Bacteroidetes* | *Bacteroidia* | *Bacteroidales* | *Porphyromonadaceae* |  |
| U00005232 | Bacteria | *Bacteroidetes* | *Bacteroidia* | *Bacteroidales* | *Porphyromonadaceae* |  |
| U00000305 | Bacteria | *Bacteroidetes* | *Bacteroidia* | *Bacteroidales* | *Porphyromonadaceae* | *Butyricimonas* |
| U00006821 | Bacteria | *Bacteroidetes* | *Bacteroidia* | *Bacteroidales* | *Porphyromonadaceae* |  |
| U01199405 | Bacteria | *Bacteroidetes* | *Bacteroidia* | *Bacteroidales* | *Porphyromonadaceae* |  |
| U00006150 | Bacteria | *Bacteroidetes* | *Bacteroidia* | *Bacteroidales* | *Porphyromonadaceae* |  |
| U00000206 | Bacteria | *Bacteroidetes* | *Bacteroidia* | *Bacteroidales* | *Porphyromonadaceae* |  |
| U01150673 | Bacteria | *Bacteroidetes* | *Bacteroidia* | *Bacteroidales* | *Porphyromonadaceae* |  |
| U00805811 | Bacteria | *Bacteroidetes* | *Bacteroidia* | *Bacteroidales* | *Porphyromonadaceae* |  |
| U01199471 | Bacteria | *Bacteroidetes* | *Bacteroidia* | *Bacteroidales* | *Porphyromonadaceae* |  |
| U00000528 | Bacteria | *Bacteroidetes* | *Bacteroidia* | *Bacteroidales* | *Porphyromonadaceae* | *Parabacteroides* |
| U00000068 | Bacteria | *Bacteroidetes* | *Bacteroidia* | *Bacteroidales* | *Porphyromonadaceae* | *Butyricimonas* |
| U00000002 | Bacteria | *Bacteroidetes* | *Bacteroidia* | *Bacteroidales* | *Bacteroidaceae* | *Bacteroides* |
| U00147566 | Bacteria | *Bacteroidetes* | *Bacteroidia* | *Bacteroidales* | *Rikenellaceae* | *Alistipes* |
| U00000394 | Bacteria | *Bacteroidetes* | *Bacteroidia* | *Bacteroidales* | *Rikenellaceae* | *Alistipes* |
| U00005559 | Bacteria | *Bacteroidetes* | *Bacteroidia* | *Bacteroidales* | *Rikenellaceae* | *Alistipes* |
| U00801961 | Bacteria |  |  |  |  |  |
| U00000126 | Bacteria | *Actinobacteria* | *Actinobacteridae* | *Bifidobacteriales* | *Bifidobacteriaceae* | *Bifidobacterium* |
| U00047397 | Bacteria | *Actinobacteria* | *Coriobacteridae* | *Coriobacteriales* | *Coriobacteriaceae* |  |
| U00000552 | Bacteria | *Bacteroidetes* | *Flavobacteria* | *Flavobacteriales* | *Flavobacteriaceae* |  |
| U00164105 | Bacteria | *Firmicutes* | *Clostridia* | *Clostridiales* | *Lachnospiraceae* |  |
| U00441706 | Bacteria | *Firmicutes* | *Clostridia* | *Clostridiales* | *IncertaeSedisXIV* | *Blautia* |
| U00118078 | Bacteria | *Firmicutes* | *Clostridia* | *Clostridiales* | *Lachnospiraceae* | *Hespellia* |
| U00119974 | Bacteria | *Firmicutes* | *Clostridia* | *Clostridiales* | *Lachnospiraceae* | *Hespellia* |
| U00104206 | Bacteria | *Firmicutes* | *Clostridia* | *Clostridiales* | *IncertaeSedisXIV* | *Blautia* |
| U00580795 | Bacteria | *Firmicutes* | *Clostridia* | *Clostridiales* |  |  |
| U01129900 | Bacteria | *Firmicutes* | *Clostridia* | *Clostridiales* | *IncertaeSedisXIV* | *Blautia* |
| U00016467 | Bacteria | *Firmicutes* | *Clostridia* | *Clostridiales* | *IncertaeSedisXIV* | *Blautia* |
| U00000367 | Bacteria | *Firmicutes* | *Clostridia* | *Clostridiales* | *IncertaeSedisXIV* | *Blautia* |
| U01148376 | Bacteria | *Firmicutes* | *Clostridia* | *Clostridiales* | *Lachnospiraceae* | *Marvinbryantia* |
| U00444002 | Bacteria | *Firmicutes* | *Clostridia* | *Clostridiales* | *Lachnospiraceae* |  |
| U01130396 | Bacteria | *Firmicutes* | *Clostridia* | *Clostridiales* | *Lachnospiraceae* |  |
| U00011582 | Bacteria | *Firmicutes* | *Clostridia* | *Clostridiales* | *Lachnospiraceae* |  |
| U00439888 | Bacteria | *Firmicutes* | *Clostridia* | *Clostridiales* | *Lachnospiraceae* |  |
| U01163617 | Bacteria | *Firmicutes* | *Clostridia* | *Clostridiales* | *Lachnospiraceae* |  |
| U00029306 | Bacteria | *Firmicutes* | *Clostridia* | *Clostridiales* | *Lachnospiraceae* |  |
| U00797985 | Bacteria | *Firmicutes* | *Clostridia* | *Clostridiales* | *Lachnospiraceae* |  |
| U01173594 | Bacteria | *Firmicutes* | *Clostridia* | *Clostridiales* | *Lachnospiraceae* |  |
| U00009282 | Bacteria | *Firmicutes* | *Clostridia* | *Clostridiales* | *Lachnospiraceae* | *Oribacterium* |
| U00165288 | Bacteria | *Firmicutes* | *Clostridia* | *Clostridiales* | *Lachnospiraceae* | *Coprococcus* |
| U00003481 | Bacteria | *Firmicutes* | *Clostridia* | *Clostridiales* | *Ruminococcaceae* | *Ruminococcus* |
| U00003548 | Bacteria | *Firmicutes* | *Clostridia* | *Clostridiales* | *Lachnospiraceae* | *Roseburia* |
| U00798472 | Bacteria | *Firmicutes* | *Clostridia* | *Clostridiales* | *Lachnospiraceae* |  |
| U00804005 | Bacteria | *Firmicutes* | *Clostridia* | *Clostridiales* | *Lachnospiraceae* |  |
| U00164814 | Bacteria | *Firmicutes* | *Clostridia* | *Clostridiales* | *Lachnospiraceae* |  |
| U00507409 | Bacteria | *Firmicutes* | *Clostridia* | *Clostridiales* | *Lachnospiraceae* |  |
| U00009952 | Bacteria | *Firmicutes* | *Clostridia* | *Clostridiales* | *Lachnospiraceae* |  |
| U00459481 | Bacteria | *Firmicutes* | *Clostridia* | *Clostridiales* |  |  |
| U00832549 | Bacteria | *Firmicutes* | *Clostridia* | *Clostridiales* | *Lachnospiraceae* | *Butyrivibrio* |
| U00013476 | Bacteria | *Firmicutes* | *Clostridia* | *Clostridiales* | *Lachnospiraceae* |  |
| U00000012 | Bacteria | *Firmicutes* | *Clostridia* | *Clostridiales* | *Lachnospiraceae* |  |
| U00808065 | Bacteria | *Firmicutes* | *Clostridia* | *Clostridiales* | *Lachnospiraceae* |  |
| U00816625 | Bacteria | *Firmicutes* | *Clostridia* | *Clostridiales* | *Lachnospiraceae* |  |
| U00164605 | Bacteria | *Firmicutes* | *Clostridia* | *Clostridiales* | *Lachnospiraceae* |  |
| U00232096 | Bacteria | *Firmicutes* | *Clostridia* | *Clostridiales* | *Lachnospiraceae* |  |
| U00031502 | Bacteria | *Firmicutes* | *Clostridia* | *Clostridiales* | *Lachnospiraceae* |  |
| U01130683 | Bacteria | *Firmicutes* | *Clostridia* | *Clostridiales* | *Lachnospiraceae* |  |
| U00000215 | Bacteria | *Firmicutes* | *Clostridia* | *Clostridiales* | *Lachnospiraceae* |  |
| U01139355 | Bacteria | *Firmicutes* | *Clostridia* | *Clostridiales* | *Lachnospiraceae* | *Roseburia* |
| U00851599 | Bacteria | *Firmicutes* | *Clostridia* | *Clostridiales* | *Lachnospiraceae* |  |
| U00071963 | Bacteria | *Firmicutes* | *Clostridia* | *Clostridiales* | *Lachnospiraceae* |  |
| U00010047 | Bacteria | *Firmicutes* | *Clostridia* | *Clostridiales* | *Lachnospiraceae* | *Coprococcus* |
| U00164135 | Bacteria |  |  |  |  |  |
| U00034050 | Bacteria | *Firmicutes* | *Clostridia* | *Clostridiales* | *Lachnospiraceae* |  |
| U01139670 | Bacteria | *Firmicutes* | *Clostridia* | *Clostridiales* | *Lachnospiraceae* |  |
| U00033670 | Bacteria | *Firmicutes* | *Clostridia* | *Clostridiales* | *Lachnospiraceae* | *Coprococcus* |
| U00033693 | Bacteria | *Firmicutes* | *Clostridia* | *Clostridiales* | *Lachnospiraceae* |  |
| U00002583 | Bacteria | *Firmicutes* | *Clostridia* | *Clostridiales* | *Lachnospiraceae* |  |
| U00044129 | Bacteria |  |  |  |  |  |
| U00042653 | Bacteria |  |  |  |  |  |
| U00855328 | Bacteria | *Firmicutes* | *Clostridia* | *Clostridiales* | *Lachnospiraceae* | *Marvinbryantia* |
| U00000939 | Bacteria | *Firmicutes* | *Clostridia* | *Clostridiales* | *Lachnospiraceae* |  |
| U00472681 | Bacteria | *Firmicutes* | *Clostridia* | *Clostridiales* | *Lachnospiraceae* |  |
| U00164196 | Bacteria | *Firmicutes* | *Clostridia* | *Clostridiales* | *Lachnospiraceae* |  |
| U00000105 | Bacteria | *Firmicutes* | *Clostridia* | *Clostridiales* | *Lachnospiraceae* |  |
| U00164256 | Bacteria | *Firmicutes* | *Clostridia* | *Clostridiales* | *Lachnospiraceae* |  |
| U00000279 | Bacteria | *Firmicutes* | *Clostridia* | *Clostridiales* |  |  |
| U00022935 | Bacteria | *Firmicutes* | *Clostridia* | *Clostridiales* |  |  |
| U00003079 | Bacteria | *Firmicutes* | *Clostridia* | *Clostridiales* | *Lachnospiraceae* | *Coprococcus* |
| U00002404 | Bacteria | *Firmicutes* | *Clostridia* | *Clostridiales* | *Ruminococcaceae* |  |
| U00804375 | Bacteria | *Firmicutes* | *Clostridia* | *Clostridiales* |  |  |
| U00443872 | Bacteria | *Firmicutes* | *Clostridia* | *Clostridiales* |  |  |
| U00000550 | Bacteria | *Firmicutes* | *Clostridia* | *Clostridiales* |  |  |
| U00000128 | Bacteria | *Firmicutes* | *Clostridia* | *Clostridiales* | *Ruminococcaceae* |  |
| U00002777 | Bacteria | *Firmicutes* | *Clostridia* | *Clostridiales* | *Ruminococcaceae* | *Oscillibacter* |
| U00001166 | Bacteria | *Firmicutes* | *Clostridia* | *Clostridiales* |  |  |
| U00002087 | Bacteria | *Firmicutes* | *Clostridia* | *Clostridiales* |  |  |
| U00008098 | Bacteria | *Firmicutes* | *Clostridia* | *Clostridiales* | *Ruminococcaceae* | *Oscillibacter* |
| U00009280 | Bacteria | *Firmicutes* | *Clostridia* | *Clostridiales* |  |  |
| U00164237 | Bacteria | *Firmicutes* | *Clostridia* | *Clostridiales* |  |  |
| U00032911 | Bacteria | *Firmicutes* | *Clostridia* | *Clostridiales* | *IncertaeSedisXI* | *Sedimentibacter* |
| U00040533 | Bacteria | *Firmicutes* | *Clostridia* | *Clostridiales* |  |  |
| U00000981 | Bacteria | *Firmicutes* | *Clostridia* |  |  |  |
| U00436292 | Bacteria | *Firmicutes* | *Clostridia* | *Clostridiales* | *Ruminococcaceae* |  |
| U00002551 | Bacteria | *Firmicutes* | *Clostridia* | *Clostridiales* | *Ruminococcaceae* | *Ruminococcus* |
| U00164297 | Bacteria | *Firmicutes* | *Clostridia* | *Clostridiales* | *Ruminococcaceae* |  |
| U00806937 | Bacteria | *Firmicutes* | *Clostridia* | *Clostridiales* | *Ruminococcaceae* |  |
| U00002723 | Bacteria | *Firmicutes* | *Clostridia* | *Clostridiales* | *Ruminococcaceae* |  |
| U00004878 | Bacteria | *Firmicutes* | *Clostridia* | *Clostridiales* | *Ruminococcaceae* |  |
| U01142982 | Bacteria | *Firmicutes* | *Clostridia* | *Clostridiales* | *Ruminococcaceae* | *Fastidiosipila* |
| U00806884 | Bacteria | *Firmicutes* | *Clostridia* | *Clostridiales* | *Ruminococcaceae* | *Ruminococcus* |
| U00810531 | Bacteria | *Firmicutes* | *Clostridia* | *Clostridiales* | *Ruminococcaceae* | *Oscillibacter* |
| U00000149 | Bacteria | *Firmicutes* | *Clostridia* | *Clostridiales* | *Ruminococcaceae* | *Oscillibacter* |
| U00004501 | Bacteria | *Firmicutes* | *Clostridia* | *Clostridiales* | *Ruminococcaceae* | *Ruminococcus* |
| U01145869 | Bacteria | *Firmicutes* | *Clostridia* | *Clostridiales* | *Ruminococcaceae* |  |
| U00211687 | Bacteria | *Firmicutes* | *Clostridia* | *Clostridiales* | *Ruminococcaceae* |  |
| U00132882 | Bacteria | *Firmicutes* | *Clostridia* | *Clostridiales* | *Ruminococcaceae* | *Oscillibacter* |
| U01206474 | Bacteria | *Firmicutes* | *Clostridia* | *Clostridiales* | *Ruminococcaceae* |  |
| U01219720 | Bacteria | *Firmicutes* | *Clostridia* | *Clostridiales* | *Ruminococcaceae* |  |
| U00800387 | Bacteria |  |  |  |  |  |
| U01210669 | Bacteria | *Firmicutes* | *Clostridia* | *Clostridiales* |  |  |
| U00000908 | Bacteria | *Firmicutes* | *Clostridia* | *Clostridiales* | *Ruminococcaceae* |  |
| U00001394 | Bacteria | *Firmicutes* | *Clostridia* | *Clostridiales* | *Ruminococcaceae* |  |
| U00460521 | Bacteria | *Firmicutes* | *Clostridia* | *Clostridiales* | *Ruminococcaceae* |  |
| U00831992 | Bacteria | *Firmicutes* | *Clostridia* | *Clostridiales* | *Ruminococcaceae* |  |
| U00204386 | Bacteria | *Firmicutes* | *Clostridia* | *Clostridiales* | *Ruminococcaceae* | *Oscillibacter* |
| U00799453 | Bacteria | *Firmicutes* | *Clostridia* | *Clostridiales* | *Ruminococcaceae* | *Fastidiosipila* |
| U01143501 | Bacteria | *Firmicutes* | *Clostridia* | *Clostridiales* | *Ruminococcaceae* | *Oscillibacter* |
| U00815791 | Bacteria | *Firmicutes* | *Clostridia* | *Clostridiales* | *Ruminococcaceae* |  |
| U00806071 | Bacteria | *Firmicutes* | *Clostridia* | *Clostridiales* | *Ruminococcaceae* | *Oscillibacter* |
| U01132907 | Bacteria | *Firmicutes* | *Clostridia* | *Clostridiales* | *Ruminococcaceae* | *Oscillibacter* |
| U00043629 | Bacteria | *Firmicutes* | *Clostridia* | *Clostridiales* | *Ruminococcaceae* | *Oscillibacter* |
| U00463337 | Bacteria | *Firmicutes* | *Clostridia* | *Clostridiales* | *Ruminococcaceae* | *Oscillibacter* |
| U00164098 | Bacteria | *Firmicutes* | *Clostridia* | *Clostridiales* | *Ruminococcaceae* |  |
| U00812994 | Bacteria | *Firmicutes* | *Clostridia* | *Clostridiales* | *Lachnospiraceae* | *Marvinbryantia* |
| U00010018 | Bacteria | *Firmicutes* | *Clostridia* | *Clostridiales* | *Ruminococcaceae* | *Oscillibacter* |
| U00164720 | Bacteria | *Firmicutes* | *Clostridia* | *Clostridiales* | *Ruminococcaceae* |  |
| U00004418 | Bacteria | *Firmicutes* | *Clostridia* | *Clostridiales* | *Ruminococcaceae* |  |
| U00208153 | Bacteria | *Firmicutes* | *Clostridia* | *Clostridiales* |  |  |
| U00000374 | Bacteria | *Firmicutes* | *Clostridia* | *Clostridiales* | *Ruminococcaceae* | *Butyricicoccus* |
| U00030870 | Bacteria | *Firmicutes* | *Clostridia* | *Clostridiales* |  |  |
| U00837797 | Bacteria | *Firmicutes* | *Clostridia* | *Clostridiales* |  |  |
| U00449804 | Bacteria | *Firmicutes* |  |  |  |  |
| U00436332 | Bacteria |  |  |  |  |  |
| U00003435 | Bacteria | *Firmicutes* |  |  |  |  |
| U00198673 | Bacteria |  |  |  |  |  |
| U00002720 | Bacteria | *Firmicutes* | *Clostridia* |  |  |  |
| U00195707 | Bacteria |  |  |  |  |  |
| U00002686 | Bacteria |  |  |  |  |  |
| U00008959 | Bacteria | *Firmicutes* | *Clostridia* |  |  |  |
| U00034351 | Bacteria |  |  |  |  |  |
| U00437009 | Bacteria |  |  |  |  |  |
| U00032486 | Bacteria |  |  |  |  |  |
| U00000999 | Bacteria |  |  |  |  |  |
| U00002201 | Bacteria |  |  |  |  |  |
| U00001891 | Bacteria |  |  |  |  |  |
| U00803665 | Bacteria |  |  |  |  |  |
| U00027111 | Bacteria |  |  |  |  |  |
| U00454157 | Bacteria |  |  |  |  |  |
| U00080174 | Bacteria | *Firmicutes* | *Clostridia* | *Clostridiales* |  |  |
| U00003296 | Bacteria |  |  |  |  |  |
| U00803302 | Bacteria |  |  |  |  |  |
| U00032208 | Bacteria |  |  |  |  |  |
| U00001116 | Bacteria |  |  |  |  |  |
| U00807704 | Bacteria | *Firmicutes* | *Clostridia* | *Clostridiales* |  |  |
| U00000272 | Bacteria |  |  |  |  |  |
| U00027329 | Bacteria |  |  |  |  |  |
| U00003420 | Bacteria | *Firmicutes* | *Clostridia* | *Clostridiales* | *Lachnospiraceae* | *Dorea* |
| U00436238 | Bacteria | *Firmicutes* | *Clostridia* | *Clostridiales* |  |  |
| U00805277 | Bacteria | *Firmicutes* | *Clostridia* | *Clostridiales* |  |  |
| U01136238 | Bacteria |  |  |  |  |  |
| U00848639 | Bacteria |  |  |  |  |  |
| U00439751 | Bacteria | *Firmicutes* | *Clostridia* | *Clostridiales* | *Lachnospiraceae* |  |
| U00004741 | Bacteria | *Firmicutes* | *Clostridia* | *Clostridiales* |  |  |
| U00004249 | Bacteria | *Firmicutes* | *Clostridia* | *Clostridiales* | *Lachnospiraceae* |  |
| U01160251 | Bacteria | *Firmicutes* | *Erysipelotrichi* | *Erysipelotrichales* | *Erysipelotrichaceae* | *Allobaculum* |
| U01166410 | Bacteria | *Firmicutes* | *Erysipelotrichi* | *Erysipelotrichales* | *Erysipelotrichaceae* | *Allobaculum* |
| U01169117 | Bacteria | *Firmicutes* | *Erysipelotrichi* | *Erysipelotrichales* | *Erysipelotrichaceae* | *Allobaculum* |
| U01180557 | Bacteria | *Firmicutes* | *Erysipelotrichi* | *Erysipelotrichales* | *Erysipelotrichaceae* | *Allobaculum* |
| U00804007 | Bacteria | *Firmicutes* | *Erysipelotrichi* | *Erysipelotrichales* | *Erysipelotrichaceae* | *Allobaculum* |
| U01143008 | Bacteria | *Firmicutes* | *Erysipelotrichi* | *Erysipelotrichales* | *Erysipelotrichaceae* | *Allobaculum* |
| U01145549 | Bacteria | *Firmicutes* | *Erysipelotrichi* | *Erysipelotrichales* | *Erysipelotrichaceae* | *Allobaculum* |
| U00808391 | Bacteria | *Firmicutes* | *Erysipelotrichi* | *Erysipelotrichales* | *Erysipelotrichaceae* | *Allobaculum* |
| U01144224 | Bacteria | *Firmicutes* | *Erysipelotrichi* | *Erysipelotrichales* | *Erysipelotrichaceae* | *Allobaculum* |
| U01214804 | Bacteria | *Firmicutes* | *Erysipelotrichi* | *Erysipelotrichales* | *Erysipelotrichaceae* | *Allobaculum* |
| U01173110 | Bacteria | *Firmicutes* | *Erysipelotrichi* | *Erysipelotrichales* | *Erysipelotrichaceae* | *Allobaculum* |
| U00305232 | Bacteria | *Firmicutes* | *Erysipelotrichi* | *Erysipelotrichales* | *Erysipelotrichaceae* | *Allobaculum* |
| U00043735 | Bacteria | *Firmicutes* | *Erysipelotrichi* | *Erysipelotrichales* | *Erysipelotrichaceae* | *Allobaculum* |
| U00000409 | Bacteria | *Firmicutes* | *Erysipelotrichi* | *Erysipelotrichales* | *Erysipelotrichaceae* | *Allobaculum* |
| U00029718 | Bacteria | *Firmicutes* | *Erysipelotrichi* | *Erysipelotrichales* | *Erysipelotrichaceae* | *Allobaculum* |
| U00007185 | Bacteria | *Firmicutes* | *Erysipelotrichi* | *Erysipelotrichales* | *Erysipelotrichaceae* | *Allobaculum* |
| U00011248 | Bacteria | *Firmicutes* | *Erysipelotrichi* | *Erysipelotrichales* | *Erysipelotrichaceae* | *Allobaculum* |
| U01156257 | Bacteria | *Firmicutes* | *Erysipelotrichi* | *Erysipelotrichales* | *Erysipelotrichaceae* | *Allobaculum* |
| U00808203 | Bacteria | *Firmicutes* | *Erysipelotrichi* | *Erysipelotrichales* | *Erysipelotrichaceae* | *Allobaculum* |
| U00003073 | Bacteria | *Firmicutes* | *Erysipelotrichi* | *Erysipelotrichales* | *Erysipelotrichaceae* | *Holdemania* |
| U00012096 | Bacteria | *Firmicutes* |  |  |  |  |
| U01152122 | Bacteria | *Tenericutes* | *Mollicutes* | *Anaeroplasmatales* | *Anaeroplasmataceae* | *Anaeroplasma* |
| U01123199 | Bacteria | *Firmicutes* | *Clostridia* | *Clostridiales* |  |  |
| U00000235 | Bacteria | *Firmicutes* | *Clostridia* | *Clostridiales* | *Veillonellaceae* | *Phascolarctobacterium* |
| U00000737 | Bacteria | *TM7* | *TM7* | *TM7* | *TM7* | *TM7_genera_incertae*  *_sedis* |
| U00822040 | Bacteria |  |  |  |  |  |
