# Supplementary material for: Structural Changes of Gut Microbiota during Berberine-Mediated Prevention of Obesity and Insulin Resistance in High-Fat Diet-Fed Rats
Source: PLoS One. 2012 Aug 3;7(8):e42529. doi: 10.1371/journal.pone.0042529 (PMC3411811; doi:10.1371/journal.pone.0042529)
Supplement: Table S3 — Univariate comparisons of the 268 key OTUs identified by RDA. (DOC) [file pone.0042529.s008.doc]

**Table S3** Univariate comparisons of the 268 key OTUs identified by RDA

| **OTU name** | **P values calculated by Mann-Whitney test** | | | | | | **Median (first quart, third quart) %** | | | |
| --- | --- | --- | --- | --- | --- | --- | --- | --- | --- | --- |
|  | **NCD**  **vs.**  **NCD+BBR** | **NCD**  **vs.**  **HFD** | **NCD**  **vs.**  **HFD+BBR** | **NCD+BBR**  **vs.**  **HFD** | **NCD+BBR**  **vs.**  **HFD+BBR** | **HFD**  **vs.**  **HFD+BBR** | **NCD** | **NCD+BBR** | **HFD** | **HFD+BBR** |
| U00436195 | 0.035 | 0.966 | 0.035 | 0.035 | na | 0.035 | 0 (0, 0.08) | 0 (0, 0) | 0 (0, 0.06) | 0 (0, 0) |
| U00101416 | 0.006 | 0.045 | 0.006 | 0.1681 | na | 0.1681 | 0.05 (0, 0.14) | 0 (0, 0) | 0 (0, 0) | 0 (0, 0) |
| U00000407 | 0.0008 | 0.0139 | 0.0002 | 0.0049 | 0.0779 | 0.0008 | 0.65 (0.49, 2.15) | 0 (0, 0.03) | 0.22 (0.13, 0.4) | 0 (0, 0) |
| U00449939 | 0.0004 | 0.7281 | 0.002 | 0.0004 | 0.0017 | 0.015 | 0 (0, 0) | 1.83 (1.2, 3.31) | 0 (0, 0.03) | 0.44 (0.3, 0.52) |
| U01131573 | 0.0036 | 1 | 0.0565 | 0.0009 | 0.0435 | 0.0371 | 0 (0, 0) | 0.11 (0.07, 0.19) | 0 (0, 0) | 0.02 (0, 0.06) |
| U00442991 | 0.2343 | 1 | 0.0009 | 0.2796 | 0.0124 | 0.0009 | 0 (0, 0) | 0 (0, 0.03) | 0 (0, 0) | 0.07 (0.02, 0.14) |
| U01131468 | 0.1681 | 0.3681 | 0.0002 | 0.5842 | 0.01 | 0.0009 | 0 (0, 0) | 0 (0, 0) | 0 (0, 0) | 0.09 (0.03, 0.27) |
| U00000076 | 0.0371 | 0.2796 | 0.0001 | 0.1996 | 0.0016 | 0.0001 | 0 (0, 0) | 0.04 (0, 0.06) | 0 (0, 0.03) | 0.89 (0.56, 1.2) |
| U00000261 | 0.0403 | 0.2343 | 0.0001 | 0.2006 | 0.0054 | 0.0002 | 0 (0, 0) | 0.03 (0, 0.37) | 0 (0, 0.04) | 0.72 (0.37, 1.12) |
| U00277049 | 0.3681 | na | 0.0149 | 0.3681 | 0.1131 | 0.0149 | 0 (0, 0) | 0 (0, 0) | 0 (0, 0) | 0.01 (0, 0.04) |
| U01140154 | 0.0008 | na | 0.0149 | 0.0008 | 0.0121 | 0.0149 | 0 (0, 0) | 0.1 (0.05, 0.17) | 0 (0, 0) | 0.01 (0, 0.04) |
| U00797917 | 0.0003 | 0.1206 | 0.0142 | 0.0001 | 0.0073 | 0.0013 | 0.02 (0, 0.11) | 1.89 (1.21, 4.12) | 0 (0, 0) | 0.55 (0.13, 0.9) |
| U00800966 | 0.0371 | 0.4818 | 0.006 | 0.2343 | 0.3681 | 0.0779 | 0.05 (0, 0.11) | 0 (0, 0) | 0 (0, 0.15) | 0 (0, 0) |
| U01131982 | 0.0005 | 0.2411 | 0.0006 | 0.0056 | 0.8942 | 0.0049 | 0.24 (0.1, 0.39) | 0 (0, 0.04) | 0.5 (0.15, 0.77) | 0 (0, 0.02) |
| U00797839 | 0.035 | 0.1826 | 0.035 | 0.006 | na | 0.006 | 0 (0, 0.05) | 0 (0, 0) | 0.06 (0, 0.15) | 0 (0, 0) |
| U00003981 | 0.035 | 0.8245 | 0.035 | 0.0779 | na | 0.0779 | 0 (0, 0.16) | 0 (0, 0) | 0 (0, 0.09) | 0 (0, 0) |
| U00001404 | 0.0002 | 0.2694 | 0.0002 | 0.006 | na | 0.006 | 0.15 (0.06, 0.23) | 0 (0, 0) | 0.07 (0, 0.15) | 0 (0, 0) |
| U00000052 | 0.0001 | 0.4272 | 0.0001 | 0.0019 | 1 | 0.0014 | 0.21 (0.13, 0.34) | 0 (0, 0) | 0.16 (0.04, 0.27) | 0 (0, 0) |
| U00000964 | 0.0001 | 0.0756 | 0.0001 | 0.0008 | na | 0.0008 | 0.24 (0.2, 0.42) | 0 (0, 0) | 0.16 (0.08, 0.2) | 0 (0, 0) |
| U00040690 | 0.006 | 0.3413 | 0.006 | 0.0779 | na | 0.0779 | 0.04 (0, 0.05) | 0 (0, 0) | 0 (0, 0.06) | 0 (0, 0) |
| U00823991 | 0.006 | 0.0237 | 0.006 | 0.3681 | na | 0.3681 | 0.02 (0, 0.04) | 0 (0, 0) | 0 (0, 0) | 0 (0, 0) |
| U00000804 | 0.1433 | 0.5005 | 0.0149 | 0.0366 | 0.1681 | 0.006 | 0.02 (0, 0.22) | 0 (0, 0) | 0.1 (0, 0.3) | 0 (0, 0) |
| U01155915 | 0.0008 | 0.0357 | 0.0008 | 0.0779 | na | 0.0779 | 0.04 (0.03, 0.07) | 0 (0, 0) | 0 (0, 0.02) | 0 (0, 0) |
| U00459850 | 0.0001 | 0.0036 | 0.0001 | 0.0008 | na | 0.0008 | 0.42 (0.23, 0.54) | 0 (0, 0) | 0.09 (0.06, 0.15) | 0 (0, 0) |
| U00798098 | 0.0008 | 0.1717 | 0.0008 | 0.035 | na | 0.035 | 0.07 (0.03, 0.16) | 0 (0, 0) | 0 (0, 0.07) | 0 (0, 0) |
| U00009170 | 0.0257 | 0.0008 | 0.0001 | 0.0139 | 0.0001 | 0.0063 | 1.02 (0.42, 2.35) | 0.35 (0.17, 0.5) | 0.09 (0.02, 0.17) | 0 (0, 0) |
| U01102153 | 0.0147 | 0.0187 | 0.006 | 1 | 0.3681 | 0.3681 | 0.04 (0, 0.06) | 0 (0, 0) | 0 (0, 0) | 0 (0, 0) |
| U00006230 | 0.0002 | 0.5953 | 0.0002 | 0.0022 | na | 0.0022 | 0.21 (0.05, 0.27) | 0 (0, 0) | 0.11 (0.01, 0.29) | 0 (0, 0) |
| U00465344 | 0.0149 | 0.3994 | 0.0149 | 0.1681 | na | 0.1681 | 0.02 (0, 0.04) | 0 (0, 0) | 0 (0, 0) | 0 (0, 0) |
| U00001202 | 0.046 | 0.1258 | 0.006 | 0.3305 | 0.3681 | 0.0779 | 0.06 (0, 0.12) | 0 (0, 0) | 0 (0, 0.02) | 0 (0, 0) |
| U00001384 | 0.0022 | 0.2847 | 0.0022 | 0.035 | na | 0.035 | 0.17 (0.01, 0.2) | 0 (0, 0) | 0 (0, 0.15) | 0 (0, 0) |
| U00802799 | 0.0001 | 0.8501 | 0.0001 | 0.0014 | 1 | 0.0014 | 0.38 (0.13, 0.84) | 0 (0, 0) | 0.38 (0.09, 0.7) | 0 (0, 0) |
| U00808453 | 0.0008 | 0.4631 | 0.0008 | 0.0149 | na | 0.0149 | 0.08 (0.03, 0.09) | 0 (0, 0) | 0.02 (0, 0.09) | 0 (0, 0) |
| U01135802 | 0.035 | 0.3681 | 0.006 | 0.1012 | 0.3127 | 0.0187 | 0 (0, 0) | 0 (0, 0.03) | 0 (0, 0) | 0.03 (0, 0.06) |
| U01199632 | 0.0063 | 0.3681 | 0.0237 | 0.0022 | 0.5624 | 0.006 | 0 (0, 0) | 0.04 (0.01, 0.05) | 0 (0, 0) | 0.03 (0, 0.06) |
| U01136954 | 0.0779 | na | 0.0149 | 0.0779 | 0.6087 | 0.0149 | 0 (0, 0) | 0 (0, 0.03) | 0 (0, 0) | 0.01 (0, 0.04) |
| U00026711 | 0.0781 | 0.0362 | 0.0002 | 0.9054 | 0.0149 | 0.006 | 0.14 (0.06, 0.19) | 0.02 (0, 0.12) | 0.05 (0, 0.08) | 0 (0, 0) |
| U00000650 | 0.0757 | 0.0539 | 0.0009 | 0.0073 | 0.0435 | 0.0004 | 1.04 (0.72, 1.2) | 0.2 (0.07, 0.8) | 2.16 (1.04, 3.61) | 0.01 (0, 0.16) |
| U00000839 | 0.0001 | 0.0058 | 0.0001 | 0.0014 | 0.3681 | 0.0008 | 0.71 (0.59, 1.22) | 0 (0, 0) | 0.24 (0.17, 0.49) | 0 (0, 0) |
| U01157930 | 0.006 | 0.1826 | 0.006 | 0.035 | na | 0.035 | 0.04 (0, 0.09) | 0 (0, 0) | 0 (0, 0.03) | 0 (0, 0) |
| U00798420 | 0.0022 | 0.0063 | 0.0022 | 0.3681 | na | 0.3681 | 0.04 (0.01, 0.05) | 0 (0, 0) | 0 (0, 0) | 0 (0, 0) |
| U00001933 | 0.0008 | 0.063 | 0.0008 | 0.0008 | na | 0.0008 | 0.06 (0.04, 0.14) | 0 (0, 0) | 0.27 (0.15, 0.56) | 0 (0, 0) |
| U00001491 | 0.273 | 0.0539 | 0.0001 | 0.162 | 0.0004 | 0.0002 | 0.71 (0.31, 0.85) | 0.42 (0.26, 0.61) | 0.27 (0.2, 0.35) | 0 (0, 0) |
| U00006476 | 0.035 | 0.2505 | 0.035 | 0.0022 | na | 0.0022 | 0 (0, 0.05) | 0 (0, 0) | 0.07 (0.01, 0.17) | 0 (0, 0) |
| U01156163 | 0.0149 | na | 0.0022 | 0.0149 | 1 | 0.0022 | 0 (0, 0) | 0.02 (0, 0.07) | 0 (0, 0) | 0.03 (0.01, 0.04) |
| U00001502 | 0.0464 | 0.7906 | 0.0008 | 0.0385 | 0.035 | 0.0008 | 0.08 (0.04, 0.14) | 0 (0, 0.05) | 0.12 (0.03, 0.18) | 0 (0, 0) |
| U00001504 | 0.0022 | 0.6065 | 0.0063 | 0.035 | 0.3681 | 0.1012 | 0.06 (0.01, 0.16) | 0 (0, 0) | 0 (0, 0.12) | 0 (0, 0) |
| U00000609 | 0.0001 | 0.6232 | 0.0001 | 0.0001 | 1 | 0.0001 | 0.93 (0.84, 1.72) | 0 (0, 0.02) | 1.11 (0.46, 1.45) | 0 (0, 0.02) |
| U00436427 | 0.0049 | 1 | 0.0014 | 0.0049 | 0.1375 | 0.0014 | 0 (0, 0) | 0.11 (0.02, 0.52) | 0 (0, 0) | 0.68 (0.25, 0.88) |
| U00009261 | 0.0002 | 0.2505 | 0.0004 | 0.0149 | 0.3681 | 0.0403 | 0.17 (0.1, 0.27) | 0 (0, 0) | 0.01 (0, 0.24) | 0 (0, 0) |
| U01136992 | 0.0001 | 0.3429 | 0.0001 | 0.006 | na | 0.006 | 0.13 (0.1, 0.28) | 0 (0, 0) | 0.08 (0, 0.16) | 0 (0, 0) |
| U01161731 | 0.0002 | na | 0.0022 | 0.0002 | 0.0485 | 0.0022 | 0 (0, 0) | 0.09 (0.04, 0.2) | 0 (0, 0) | 0.03 (0.01, 0.05) |
| U00804243 | 0.0001 | 0.0184 | 0.0002 | 0.0002 | 0.0113 | 0.0013 | 0 (0, 0) | 2.89 (1.47, 7.94) | 0.08 (0.01, 0.15) | 1.02 (0.78, 1.51) |
| U00832647 | 0.006 | na | 0.035 | 0.006 | 0.1575 | 0.035 | 0 (0, 0) | 0.07 (0, 0.17) | 0 (0, 0) | 0 (0, 0.04) |
| U00000497 | 0.0004 | 0.2723 | 0.0006 | 0.0014 | 0.6704 | 0.0025 | 0.67 (0.32, 1.56) | 0 (0, 0) | 0.44 (0.19, 0.69) | 0 (0, 0) |
| U00013412 | 0.0154 | 0.387 | 0.0001 | 0.0063 | 0.8499 | 0.0001 | 0 (0, 0.02) | 1.8 (0.05, 3.71) | 0 (0, 0) | 1.6 (1.06, 2.29) |
| U00000886 | 0.0001 | 0.3073 | 0.0001 | 0.0014 | 0.3681 | 0.0008 | 0.27 (0.17, 0.41) | 0 (0, 0) | 0.18 (0.1, 0.29) | 0 (0, 0) |
| U01143292 | 0.0022 | 0.0022 | 0.0022 | na | na | na | 0.07 (0.01, 0.29) | 0 (0, 0) | 0 (0, 0) | 0 (0, 0) |
| U00807079 | 0.0002 | 0.1012 | 0.0004 | 0.0015 | 0.0257 | 0.0121 | 0 (0, 0) | 0.6 (0.49, 1.62) | 0 (0, 0.15) | 0.24 (0.18, 0.4) |
| U00803188 | 0.0002 | 0.5452 | 0.0002 | 0.0002 | na | 0.0002 | 0.21 (0.12, 0.28) | 0 (0, 0) | 0.23 (0.17, 0.41) | 0 (0, 0) |
| U00008782 | 0.0008 | 0.4668 | 0.0008 | 0.006 | na | 0.006 | 0.06 (0.04, 0.09) | 0 (0, 0) | 0.1 (0, 0.22) | 0 (0, 0) |
| U00005346 | 0.0073 | 0.2411 | 0.0073 | 0.0057 | 0.5205 | 0.0073 | 0.43 (0.29, 0.82) | 2.57 (1.38, 2.87) | 0.21 (0.09, 0.42) | 2.23 (1.02, 2.41) |
| U00004086 | 0.0002 | 0.2088 | 0.0008 | 0.0002 | 0.0036 | 0.0639 | 0.06 (0.01, 0.13) | 0.92 (0.76, 1.97) | 0.12 (0.04, 0.27) | 0.3 (0.2, 0.55) |
| U00001406 | 0.0001 | 0.0172 | 0.0001 | 0.026 | 0.035 | 0.0008 | 0.39 (0.2, 1.09) | 0 (0, 0.05) | 0.11 (0.03, 0.22) | 0 (0, 0) |
| U00030880 | 0.035 | 0.0002 | 0.035 | 0.0001 | na | 0.0001 | 0 (0, 0.04) | 0 (0, 0) | 0.29 (0.25, 0.34) | 0 (0, 0) |
| U01130438 | 0.1681 | na | 0.006 | 0.1681 | 0.186 | 0.006 | 0 (0, 0) | 0 (0, 0) | 0 (0, 0) | 0.02 (0, 0.04) |
| U01131459 | 0.0779 | na | 0.0149 | 0.0779 | 0.8645 | 0.0149 | 0 (0, 0) | 0 (0, 0.04) | 0 (0, 0) | 0.01 (0, 0.03) |
| U00436448 | 0.348 | 0.8521 | 0.0002 | 0.4426 | 0.0012 | 0.0004 | 0 (0, 0.03) | 0.02 (0, 0.05) | 0 (0, 0.03) | 0.17 (0.15, 0.26) |
| U00474862 | na | na | 0.006 | na | 0.006 | 0.006 | 0 (0, 0) | 0 (0, 0) | 0 (0, 0) | 0.02 (0, 0.04) |
| U00450938 | 0.0001 | 0.8501 | 0.0001 | 0.0004 | 1 | 0.0004 | 0.38 (0.33, 0.62) | 0 (0, 0) | 0.52 (0.16, 1.72) | 0 (0, 0) |
| U00009763 | 0.0001 | 0.4274 | 0.0001 | 0.0001 | 1 | 0.0001 | 1.02 (0.63, 1.24) | 0 (0, 0) | 1.22 (0.66, 3.32) | 0 (0, 0) |
| U01155495 | 0.0002 | 0.9389 | 0.0002 | 0.0149 | na | 0.0149 | 0.05 (0.04, 0.09) | 0 (0, 0) | 0.04 (0, 0.14) | 0 (0, 0) |
| U00798694 | 0.035 | 0.5907 | 0.035 | 0.0149 | na | 0.0149 | 0 (0, 0.04) | 0 (0, 0) | 0.01 (0, 0.07) | 0 (0, 0) |
| U00001388 | 0.0002 | 0.1015 | 0.0002 | 0.0002 | 0.5205 | 0.0002 | 0.28 (0.1, 0.33) | 3.3 (2.94, 3.92) | 0.1 (0.01, 0.15) | 4.56 (2.59, 5.98) |
| U01185184 | 0.0022 | na | 0.035 | 0.0022 | 0.1426 | 0.035 | 0 (0, 0) | 0.05 (0.01, 0.08) | 0 (0, 0) | 0 (0, 0.04) |
| U00802360 | 0.1236 | 0.0111 | 0.035 | 0.0014 | 0.3681 | 0.0008 | 0 (0, 0.11) | 0 (0, 0) | 0.3 (0.12, 0.73) | 0 (0, 0) |
| U01162782 | 0.035 | 0.353 | 0.035 | 0.006 | na | 0.006 | 0 (0, 0.07) | 0 (0, 0) | 0.05 (0, 0.13) | 0 (0, 0) |
| U00002709 | 0.0001 | 0.3847 | 0.0001 | 0.0002 | 0.1681 | 0.0006 | 1.19 (0.79, 1.75) | 0 (0, 0) | 1.62 (0.88, 2.38) | 0 (0, 0) |
| U00005232 | 0.0033 | 0.301 | 0.0008 | 0.0298 | 0.3681 | 0.006 | 0.06 (0.05, 0.15) | 0 (0, 0) | 0.04 (0, 0.08) | 0 (0, 0) |
| U00000305 | 0.0008 | 0.9395 | 0.0002 | 0.0154 | 0.0779 | 0.0022 | 0.37 (0.12, 0.59) | 0 (0, 0.02) | 0.42 (0.05, 0.57) | 0 (0, 0) |
| U00006821 | 0.0001 | 0.0376 | 0.0001 | 0.0014 | 1 | 0.0014 | 0.9 (0.65, 1.15) | 0 (0, 0) | 0.24 (0.11, 0.7) | 0 (0, 0) |
| U01199405 | 0.006 | 0.2302 | 0.006 | 0.0779 | na | 0.0779 | 0.04 (0, 0.05) | 0 (0, 0) | 0 (0, 0.03) | 0 (0, 0) |
| U00006150 | 0.0001 | 0.3445 | 0.0001 | 0.0008 | 0.3681 | 0.0019 | 0.17 (0.14, 0.24) | 0 (0, 0) | 0.08 (0.03, 0.34) | 0 (0, 0) |
| U00000206 | 0.0035 | 0.1618 | 0.0001 | 0.18 | 0.006 | 0.0008 | 0.38 (0.18, 0.54) | 0.05 (0, 0.11) | 0.16 (0.05, 0.29) | 0 (0, 0) |
| U01150673 | 0.0149 | 0.1433 | 0.0149 | 0.1681 | na | 0.1681 | 0.02 (0, 0.07) | 0 (0, 0) | 0 (0, 0) | 0 (0, 0) |
| U00805811 | 0.0001 | 0.9097 | 0.0001 | 0.0001 | 0.3681 | 0.0001 | 0.49 (0.32, 0.55) | 0 (0, 0) | 0.49 (0.18, 1.28) | 0 (0, 0) |
| U01199471 | 0.0149 | 0.5503 | 0.0149 | 0.0779 | na | 0.0779 | 0.02 (0, 0.05) | 0 (0, 0) | 0 (0, 0.02) | 0 (0, 0) |
| U00000528 | 0.0022 | 0.0779 | 0.0022 | 0.0357 | 0.54 | 0.0899 | 0 (0, 0) | 0.11 (0.01, 0.22) | 0 (0, 0.04) | 0.04 (0.01, 0.16) |
| U00000068 | 0.0298 | 0.2755 | 0.006 | 0.1799 | 0.3681 | 0.035 | 0.04 (0, 0.09) | 0 (0, 0) | 0 (0, 0.05) | 0 (0, 0) |
| U00000002 | 0.031 | 0.4238 | 0.0002 | 0.0951 | 0.0028 | 0.0002 | 0.05 (0.03, 0.05) | 0.13 (0.06, 0.37) | 0.06 (0.01, 0.08) | 0.93 (0.67, 1.29) |
| U00147566 | 0.035 | na | 0.0149 | 0.035 | 0.967 | 0.0149 | 0 (0, 0) | 0 (0, 0.06) | 0 (0, 0) | 0.02 (0, 0.06) |
| U00000394 | 0.0001 | 0.1681 | 0.0002 | 0.0001 | 0.6776 | 0.0001 | 0 (0, 0) | 0.36 (0.25, 0.41) | 0 (0, 0) | 0.37 (0.29, 0.55) |
| U00005559 | 0.0025 | 0.0435 | 0.0001 | 0.5349 | 0.1236 | 0.0503 | 0.2 (0.1, 0.29) | 0 (0, 0.04) | 0.01 (0, 0.16) | 0 (0, 0) |
| U00801961 | 0.0005 | 0.139 | 0.0001 | 0.0549 | 0.5036 | 0.0147 | 0.33 (0.2, 0.5) | 0 (0, 0) | 0.16 (0, 0.4) | 0 (0, 0) |
| U00000126 | 0.0049 | 0.0405 | 0.1928 | 0.0004 | 0.0105 | 0.0019 | 0.12 (0.02, 0.97) | 0 (0, 0) | 1.77 (0.48, 4.68) | 0.04 (0.01, 0.1) |
| U00047397 | na | 0.3681 | 0.006 | 0.3681 | 0.006 | 0.0187 | 0 (0, 0) | 0 (0, 0) | 0 (0, 0) | 0.03 (0, 0.05) |
| U00000552 | 0.0115 | 0.2674 | 0.1133 | 0.0014 | 0.1613 | 0.0125 | 0.02 (0, 0.1) | 0.2 (0.09, 0.54) | 0 (0, 0.03) | 0.12 (0.03, 0.16) |
| U00164105 | 0.0022 | 0.0756 | 0.0022 | 0.0779 | na | 0.0779 | 0.04 (0.01, 0.07) | 0 (0, 0) | 0 (0, 0.02) | 0 (0, 0) |
| U00441706 | 0.0149 | 0.1681 | 0.0022 | 0.2311 | 0.6962 | 0.0898 | 0 (0, 0) | 0.02 (0, 0.06) | 0 (0, 0) | 0.04 (0.01, 0.07) |
| U00118078 | 0.006 | 0.006 | 0.006 | na | na | na | 0.05 (0, 0.11) | 0 (0, 0) | 0 (0, 0) | 0 (0, 0) |
| U00119974 | 0.3681 | na | 0.0022 | 0.3681 | 0.0169 | 0.0022 | 0 (0, 0) | 0 (0, 0) | 0 (0, 0) | 0.06 (0.01, 0.15) |
| U00104206 | 0.0766 | 0.5842 | 0.0012 | 0.1984 | 0.0781 | 0.0049 | 0 (0, 0) | 0.01 (0, 0.08) | 0 (0, 0) | 0.12 (0.07, 0.28) |
| U00580795 | 0.0518 | 0.4509 | 0.0002 | 0.2755 | 0.0168 | 0.0041 | 0 (0, 0.02) | 0.06 (0, 0.1) | 0 (0, 0.04) | 0.15 (0.11, 0.28) |
| U01129900 | 0.035 | 0.1681 | 0.0008 | 0.4283 | 0.0385 | 0.0138 | 0 (0, 0) | 0 (0, 0.04) | 0 (0, 0) | 0.1 (0.04, 0.13) |
| U00016467 | 0.3681 | na | 0.0008 | 0.3681 | 0.0019 | 0.0008 | 0 (0, 0) | 0 (0, 0) | 0 (0, 0) | 0.31 (0.09, 0.39) |
| U00000367 | 0.0025 | 0.0022 | 0.0002 | 0.1212 | 0.0211 | 0.0036 | 0.06 (0.04, 0.17) | 1.45 (1.07, 3.15) | 0.5 (0.37, 1.81) | 6.22 (2.99, 13.15) |
| U01148376 | 0.0149 | 0.1984 | 0.0149 | 0.1681 | na | 0.1681 | 0.01 (0, 0.07) | 0 (0, 0) | 0 (0, 0) | 0 (0, 0) |
| U00444002 | 0.0149 | 0.8645 | 0.0149 | 0.0779 | na | 0.0779 | 0.02 (0, 0.05) | 0 (0, 0) | 0 (0, 0.06) | 0 (0, 0) |
| U01130396 | 0.0022 | 0.0518 | 0.0105 | 0.1681 | 0.3681 | 0.5842 | 0.03 (0.01, 0.04) | 0 (0, 0) | 0 (0, 0) | 0 (0, 0) |
| U00011582 | 0.0063 | 0.2114 | 0.0049 | 0.0503 | 1 | 0.0403 | 0.1 (0.01, 0.36) | 0 (0, 0) | 0.02 (0, 0.09) | 0 (0, 0) |
| U00439888 | 0.0149 | 0.9684 | 0.0149 | 0.006 | na | 0.006 | 0.02 (0, 0.32) | 0 (0, 0) | 0.06 (0, 0.25) | 0 (0, 0) |
| U01163617 | 0.0002 | 0.0002 | 0.0002 | na | na | na | 0.09 (0.05, 0.15) | 0 (0, 0) | 0 (0, 0) | 0 (0, 0) |
| U00029306 | 0.0001 | 0.0001 | 0.0001 | na | na | na | 0.64 (0.48, 1.11) | 0 (0, 0) | 0 (0, 0) | 0 (0, 0) |
| U00797985 | 0.0001 | 0.0036 | 0.0001 | 0.0008 | na | 0.0008 | 0.94 (0.86, 1.14) | 0 (0, 0) | 0.13 (0.08, 0.26) | 0 (0, 0) |
| U01173594 | 0.0149 | 0.1131 | 0.0149 | 0.3681 | na | 0.3681 | 0.01 (0, 0.04) | 0 (0, 0) | 0 (0, 0) | 0 (0, 0) |
| U00009282 | 0.6065 | 0.0578 | 0.0154 | 0.0227 | 0.3517 | 0.001 | 0.1 (0.01, 0.14) | 0 (0, 0.17) | 0.24 (0.07, 0.36) | 0 (0, 0.02) |
| U00165288 | 0.006 | 0.5522 | 0.006 | 0.0149 | na | 0.0149 | 0.04 (0, 0.04) | 0 (0, 0) | 0.02 (0, 0.28) | 0 (0, 0) |
| U00003481 | 0.0002 | 0.0043 | 0.002 | 0.1008 | 0.9569 | 0.2311 | 0.86 (0.29, 1.19) | 0 (0, 0) | 0.03 (0, 0.25) | 0 (0, 0) |
| U00003548 | 0.0022 | 0.0022 | 0.0022 | na | na | na | 0.14 (0.03, 0.15) | 0 (0, 0) | 0 (0, 0) | 0 (0, 0) |
| U00798472 | 0.0001 | 0.0001 | 0.0001 | na | 0.3681 | 0.3681 | 0.23 (0.13, 0.42) | 0 (0, 0) | 0 (0, 0) | 0 (0, 0) |
| U00804005 | 0.0149 | 0.0822 | 0.0403 | 0.0008 | 0.3681 | 0.0014 | 0.02 (0, 0.05) | 0 (0, 0) | 0.16 (0.04, 0.43) | 0 (0, 0) |
| U00164814 | 0.0503 | 0.2674 | 0.0149 | 0.2796 | 0.3681 | 0.0779 | 0.02 (0, 0.09) | 0 (0, 0) | 0 (0, 0.02) | 0 (0, 0) |
| U00507409 | 0.0008 | 0.1403 | 0.0032 | 0.0001 | 0.1681 | 0.0002 | 0.09 (0.05, 0.27) | 0 (0, 0) | 0.3 (0.16, 0.36) | 0 (0, 0) |
| U00009952 | 0.006 | 0.006 | 0.006 | na | na | na | 0.06 (0, 0.16) | 0 (0, 0) | 0 (0, 0) | 0 (0, 0) |
| U00459481 | 0.0022 | 0.3681 | 0.0002 | 0.0105 | 0.3239 | 0.0016 | 0 (0, 0) | 0.13 (0.01, 0.41) | 0 (0, 0) | 0.19 (0.13, 0.58) |
| U00832549 | 0.3219 | 0.062 | 0.0298 | 0.0022 | 0.0934 | 0.0007 | 0.04 (0, 0.1) | 0.01 (0, 0.04) | 0.14 (0.08, 0.2) | 0 (0, 0) |
| U00013476 | 0.1697 | 0.0112 | 0.0022 | 0.0008 | 0.0779 | 0.0001 | 0.06 (0.01, 0.13) | 0 (0, 0.05) | 1.26 (0.73, 1.4) | 0 (0, 0) |
| U00000012 | 0.301 | 0.341 | 0.0019 | 0.7869 | 0.0187 | 0.0063 | 0.15 (0.03, 0.52) | 0.05 (0, 0.13) | 0.07 (0.01, 0.16) | 0 (0, 0) |
| U00808065 | 0.0002 | 0.0185 | 0.0002 | 0.035 | na | 0.035 | 0.12 (0.05, 0.23) | 0 (0, 0) | 0 (0, 0.07) | 0 (0, 0) |
| U00816625 | 0.0149 | 1 | 0.0149 | 0.0149 | na | 0.0149 | 0.01 (0, 0.12) | 0 (0, 0) | 0.02 (0, 0.08) | 0 (0, 0) |
| U00164605 | 0.0116 | 0.0022 | 0.0022 | 0.1681 | 0.1681 | na | 0.26 (0.04, 0.46) | 0 (0, 0) | 0 (0, 0) | 0 (0, 0) |
| U00232096 | 0.0149 | 0.0149 | 0.0149 | na | na | na | 0.01 (0, 0.04) | 0 (0, 0) | 0 (0, 0) | 0 (0, 0) |
| U00031502 | 0.0803 | 0.2755 | 0.006 | 0.3278 | 0.1681 | 0.035 | 0.09 (0, 0.18) | 0 (0, 0) | 0 (0, 0.07) | 0 (0, 0) |
| U01130683 | 0.006 | 0.0298 | 0.0147 | 0.3681 | 0.3681 | 1 | 0.04 (0, 0.12) | 0 (0, 0) | 0 (0, 0) | 0 (0, 0) |
| U00000215 | 0.0002 | 0.3681 | 0.0002 | 0.0004 | 0.5966 | 0.0007 | 0 (0, 0) | 0.42 (0.26, 0.93) | 0 (0, 0) | 0.46 (0.1, 0.72) |
| U01139355 | 0.0149 | 0.0149 | 0.0149 | na | na | na | 0.02 (0, 0.06) | 0 (0, 0) | 0 (0, 0) | 0 (0, 0) |
| U00851599 | 0.0149 | 0.1426 | 0.0149 | 0.006 | na | 0.006 | 0.02 (0, 0.05) | 0 (0, 0) | 0.13 (0, 0.28) | 0 (0, 0) |
| U00071963 | 0.3681 | na | 0.0008 | 0.3681 | 0.0116 | 0.0008 | 0 (0, 0) | 0 (0, 0) | 0 (0, 0) | 0.03 (0.02, 0.04) |
| U00010047 | 0.3845 | 0.2413 | 0.0013 | 0.0889 | 0.0252 | 0.0004 | 0.28 (0.13, 0.95) | 0.16 (0.06, 0.52) | 0.64 (0.37, 1.21) | 0.04 (0.04, 0.05) |
| U00164135 | 0.0022 | 0.0022 | 0.0022 | na | na | na | 0.05 (0.01, 0.06) | 0 (0, 0) | 0 (0, 0) | 0 (0, 0) |
| U00034050 | 0.006 | 0.9054 | 0.0147 | 0.0149 | 0.3681 | 0.0403 | 0.06 (0, 0.15) | 0 (0, 0) | 0.03 (0, 0.23) | 0 (0, 0) |
| U01139670 | 0.0008 | 0.0008 | 0.0008 | na | na | na | 0.15 (0.09, 0.33) | 0 (0, 0) | 0 (0, 0) | 0 (0, 0) |
| U00033670 | 0.006 | 0.1354 | 0.006 | 0.1681 | na | 0.1681 | 0.05 (0, 0.12) | 0 (0, 0) | 0 (0, 0) | 0 (0, 0) |
| U00033693 | 0.0022 | 0.0049 | 0.0022 | 0.3681 | na | 0.3681 | 0.12 (0.01, 0.16) | 0 (0, 0) | 0 (0, 0) | 0 (0, 0) |
| U00002583 | 0.2411 | 0.0376 | 0.0004 | 0.0373 | 0.01 | 0.0008 | 0.18 (0.12, 0.3) | 0.09 (0.06, 0.25) | 0.48 (0.3, 0.72) | 0 (0, 0.02) |
| U00044129 | 0.006 | 0.0298 | 0.006 | 0.3681 | na | 0.3681 | 0.04 (0, 0.16) | 0 (0, 0) | 0 (0, 0) | 0 (0, 0) |
| U00042653 | 0.0023 | 0.0001 | 0.0001 | 0.0779 | 0.0779 | na | 0.19 (0.14, 0.32) | 0 (0, 0.07) | 0 (0, 0) | 0 (0, 0) |
| U00855328 | 0.0149 | 0.1206 | 0.0149 | 0.1681 | na | 0.1681 | 0.01 (0, 0.16) | 0 (0, 0) | 0 (0, 0) | 0 (0, 0) |
| U00000939 | 0.4846 | 0.6704 | 0.0049 | 0.1799 | 0.0077 | 0.0016 | 0 (0, 0) | 0 (0, 0.04) | 0 (0, 0) | 0.1 (0.05, 0.42) |
| U00472681 | 0.0007 | 0.035 | 0.6389 | 0.0001 | 0.0103 | 0.035 | 0 (0, 0.03) | 0.34 (0.15, 0.59) | 0 (0, 0) | 0 (0, 0.14) |
| U00164196 | 0.035 | 0.5005 | 0.035 | 0.0022 | na | 0.0022 | 0 (0, 0.4) | 0 (0, 0) | 0.08 (0.01, 0.44) | 0 (0, 0) |
| U00000105 | 0.0003 | 0.146 | 0.0099 | 0.0002 | 0.7913 | 0.003 | 0.04 (0.01, 0.07) | 1.7 (1.11, 2.08) | 0 (0, 0.03) | 1.42 (0.17, 2.54) |
| U00164256 | 0.0133 | 0.6776 | 0.0004 | 0.0464 | 0.7815 | 0.0054 | 0.24 (0.08, 0.31) | 0.01 (0, 0.08) | 0.13 (0.08, 0.31) | 0.03 (0, 0.04) |
| U00000279 | 0.966 | 0.035 | 0.0052 | 0.035 | 0.0065 | 0.0001 | 0 (0, 0.03) | 0 (0, 0.08) | 0 (0, 0) | 0.15 (0.09, 0.54) |
| U00022935 | 0.0147 | 0.3681 | 0.0001 | 0.006 | 0.0027 | 0.0001 | 0 (0, 0) | 0.1 (0, 0.17) | 0 (0, 0) | 0.35 (0.25, 0.49) |
| U00003079 | 0.006 | 0.9054 | 0.0147 | 0.0149 | 0.3681 | 0.0403 | 0.06 (0, 0.12) | 0 (0, 0) | 0.06 (0, 0.17) | 0 (0, 0) |
| U00002404 | 0.011 | 0.4727 | 0.0227 | 0.0107 | 0.3413 | 0.0288 | 0.17 (0.14, 0.19) | 0.04 (0, 0.06) | 0.22 (0.11, 0.39) | 0 (0, 0.02) |
| U00804375 | 0.006 | 0.84 | 0.0147 | 0.035 | 0.3681 | 0.1012 | 0.04 (0, 0.05) | 0 (0, 0) | 0 (0, 0.06) | 0 (0, 0) |
| U00443872 | 0.0149 | 0.8361 | 0.0149 | 0.035 | na | 0.035 | 0.01 (0, 0.09) | 0 (0, 0) | 0 (0, 0.17) | 0 (0, 0) |
| U00000550 | 0.1236 | 0.3681 | 0.0133 | 0.035 | 0.2847 | 0.0022 | 0 (0, 0) | 0 (0, 0.06) | 0 (0, 0) | 0.02 (0.01, 0.18) |
| U00000128 | 0.006 | 0.006 | 0.006 | na | na | na | 0.19 (0, 1.08) | 0 (0, 0) | 0 (0, 0) | 0 (0, 0) |
| U00002777 | 0.1842 | 0.2123 | 0.0605 | 0.0521 | 0.6569 | 0.0277 | 0.11 (0.08, 0.19) | 0.03 (0, 0.2) | 0.21 (0.13, 0.57) | 0 (0, 0.08) |
| U00001166 | 0.0008 | 0.0008 | 0.0008 | na | na | na | 0.08 (0.04, 0.09) | 0 (0, 0) | 0 (0, 0) | 0 (0, 0) |
| U00002087 | 0.0008 | 0.2574 | 0.0008 | 0.035 | na | 0.035 | 0.06 (0.04, 0.16) | 0 (0, 0) | 0 (0, 0.08) | 0 (0, 0) |
| U00008098 | 0.8501 | 0.162 | 0.0003 | 0.162 | 0.0038 | 0.0007 | 0.44 (0.41, 0.5) | 0.5 (0.17, 0.57) | 0.67 (0.4, 1.08) | 0 (0, 0.04) |
| U00009280 | 0.8245 | 0.3964 | 0.0005 | 0.1996 | 0.0002 | 0.001 | 0 (0, 0.05) | 0 (0, 0.05) | 0.03 (0, 0.11) | 1.03 (0.51, 1.81) |
| U00164237 | 1 | 0.2796 | 0.0005 | 0.2796 | 0.0005 | 0.0049 | 0 (0, 0) | 0 (0, 0) | 0 (0, 0.03) | 0.12 (0.09, 0.16) |
| U00032911 | 0.1681 | na | 0.0149 | 0.1681 | 0.3077 | 0.0149 | 0 (0, 0) | 0 (0, 0) | 0 (0, 0) | 0.01 (0, 0.04) |
| U00040533 | 0.0149 | 0.1681 | 0.0022 | 0.0837 | 0.8148 | 0.0184 | 0 (0, 0) | 0.02 (0, 0.09) | 0 (0, 0) | 0.04 (0.01, 0.05) |
| U00000981 | 0.0009 | 0.7913 | 0.0376 | 0.0029 | 0.0029 | 0.3256 | 0.28 (0.14, 0.32) | 0 (0, 0.04) | 0.19 (0.09, 0.47) | 0.13 (0.09, 0.17) |
| U00436292 | 0.006 | 0.4515 | 0.0147 | 0.0149 | 0.3681 | 0.0503 | 0.04 (0, 0.15) | 0 (0, 0) | 0.01 (0, 0.05) | 0 (0, 0) |
| U00002551 | 0.0014 | 0.0025 | 0.0056 | 1 | 1 | 1 | 0.09 (0.08, 0.39) | 0 (0, 0) | 0 (0, 0) | 0 (0, 0) |
| U00164297 | 0.0237 | 0.6065 | 0.006 | 0.0403 | 0.3681 | 0.0149 | 0.04 (0, 0.26) | 0 (0, 0) | 0.06 (0, 0.19) | 0 (0, 0) |
| U00806937 | 0.0149 | 0.0149 | 0.0149 | na | na | na | 0.08 (0, 0.23) | 0 (0, 0) | 0 (0, 0) | 0 (0, 0) |
| U00002723 | 0.0007 | 0.0016 | 0.0002 | 1 | 0.3681 | 0.3681 | 0.13 (0.06, 0.23) | 0 (0, 0) | 0 (0, 0) | 0 (0, 0) |
| U00004878 | 0.006 | 0.2302 | 0.006 | 0.0779 | na | 0.0779 | 0.02 (0, 0.07) | 0 (0, 0) | 0 (0, 0.03) | 0 (0, 0) |
| U01142982 | 0.0008 | 0.0008 | 0.0008 | na | na | na | 0.1 (0.04, 0.14) | 0 (0, 0) | 0 (0, 0) | 0 (0, 0) |
| U00806884 | 0.0022 | 0.0022 | 0.0022 | na | na | na | 0.04 (0.01, 0.1) | 0 (0, 0) | 0 (0, 0) | 0 (0, 0) |
| U00810531 | 0.006 | 0.006 | 0.006 | na | na | na | 0.03 (0, 0.12) | 0 (0, 0) | 0 (0, 0) | 0 (0, 0) |
| U00000149 | 0.0046 | 0.384 | 0.0005 | 0.132 | 0.0503 | 0.0033 | 0.28 (0.1, 0.37) | 0.02 (0, 0.05) | 0.1 (0.03, 0.29) | 0 (0, 0) |
| U00004501 | 0.0022 | 0.0022 | 0.0022 | na | na | na | 0.09 (0.01, 0.32) | 0 (0, 0) | 0 (0, 0) | 0 (0, 0) |
| U01145869 | na | 0.3681 | 0.0022 | 0.3681 | 0.0022 | 0.0082 | 0 (0, 0) | 0 (0, 0) | 0 (0, 0) | 0.03 (0.01, 0.06) |
| U00211687 | 0.0147 | 1 | 0.0237 | 0.0147 | 0.7253 | 0.0187 | 0 (0, 0) | 0.05 (0, 0.08) | 0 (0, 0) | 0.03 (0, 0.06) |
| U00132882 | 0.001 | 0.0027 | 0.0001 | 0.7253 | 0.0147 | 0.0147 | 0.41 (0.21, 0.49) | 0.04 (0, 0.15) | 0.07 (0, 0.17) | 0 (0, 0) |
| U01206474 | 0.0068 | 0.1681 | 0.0283 | 0.0008 | 0.2378 | 0.0022 | 0 (0, 0) | 0.04 (0.03, 0.1) | 0 (0, 0) | 0.04 (0.01, 0.04) |
| U01219720 | 1 | 0.3681 | 0.0133 | 0.3681 | 0.0212 | 0.0022 | 0 (0, 0) | 0 (0, 0) | 0 (0, 0) | 0.02 (0.01, 0.03) |
| U00800387 | 0.0002 | 0.0016 | 0.0002 | 0.3681 | na | 0.3681 | 0.12 (0.06, 0.45) | 0 (0, 0) | 0 (0, 0) | 0 (0, 0) |
| U01210669 | 0.2343 | 0.3681 | 0.0049 | 0.0779 | 0.1963 | 0.0022 | 0 (0, 0) | 0 (0, 0.03) | 0 (0, 0) | 0.04 (0.01, 0.07) |
| U00000908 | 0.0002 | 0.0046 | 0.0003 | 0.0246 | 0.1826 | 0.1604 | 2.08 (0.75, 2.55) | 0.04 (0.01, 0.05) | 0.24 (0.1, 0.49) | 0.09 (0.05, 0.16) |
| U00001394 | 0.0091 | 0.0012 | 0.0002 | 0.0004 | 0.7337 | 0.0002 | 0.69 (0.56, 1.67) | 4.47 (2.25, 6.23) | 0.02 (0, 0.15) | 4.02 (3.57, 6.79) |
| U00460521 | 0.0735 | 0.006 | 0.0088 | 0.0002 | 0.5205 | 0.0001 | 0.04 (0, 0.05) | 0.13 (0.04, 0.23) | 0 (0, 0) | 0.16 (0.08, 0.24) |
| U00831992 | 0.1066 | 0.1681 | 0.0139 | 0.0022 | 0.384 | 0.0001 | 0 (0, 0) | 0.05 (0.01, 0.09) | 0 (0, 0) | 0.06 (0.04, 0.12) |
| U00204386 | 0.006 | 0.006 | 0.006 | na | na | na | 0.09 (0, 0.13) | 0 (0, 0) | 0 (0, 0) | 0 (0, 0) |
| U00799453 | 0.0435 | 0.3256 | 0.0002 | 0.0107 | 0.006 | 0.0002 | 0.19 (0.1, 0.34) | 0.07 (0, 0.11) | 0.4 (0.25, 0.77) | 0 (0, 0) |
| U01143501 | 0.3681 | na | 0.0149 | 0.3681 | 0.0623 | 0.0149 | 0 (0, 0) | 0 (0, 0) | 0 (0, 0) | 0.01 (0, 0.05) |
| U00815791 | 0.3681 | na | 0.0022 | 0.3681 | 0.0063 | 0.0022 | 0 (0, 0) | 0 (0, 0) | 0 (0, 0) | 0.24 (0.01, 0.35) |
| U00806071 | 0.0063 | 0.1697 | 0.0022 | 0.2796 | 0.3681 | 0.0779 | 0.05 (0.01, 0.13) | 0 (0, 0) | 0 (0, 0.02) | 0 (0, 0) |
| U01132907 | 0.0187 | 0.0237 | 0.006 | 1 | 0.3681 | 0.3681 | 0.04 (0, 0.09) | 0 (0, 0) | 0 (0, 0) | 0 (0, 0) |
| U00043629 | 1 | 0.5907 | 0.0002 | 0.7721 | 0.0009 | 0.0002 | 0 (0, 0.05) | 0 (0, 0.04) | 0.02 (0, 0.1) | 0.82 (0.5, 1.25) |
| U00463337 | 0.0172 | 0.4631 | 0.0008 | 0.1206 | 0.1681 | 0.0149 | 0.06 (0.04, 0.28) | 0 (0, 0) | 0.02 (0, 0.29) | 0 (0, 0) |
| U00164098 | 0.9569 | 0.2838 | 0.0006 | 0.376 | 0.0008 | 0.0185 | 0 (0, 0) | 0 (0, 0) | 0 (0, 0.04) | 0.13 (0.09, 0.31) |
| U00812994 | 0.006 | 0.0565 | 0.006 | 0.3681 | na | 0.3681 | 0.04 (0, 0.05) | 0 (0, 0) | 0 (0, 0) | 0 (0, 0) |
| U00010018 | 0.0009 | 0.014 | 0.0004 | 0.2025 | 0.3343 | 0.2712 | 0.21 (0.16, 0.42) | 0.03 (0, 0.08) | 0.1 (0.04, 0.15) | 0.06 (0.03, 0.1) |
| U00164720 | 0.0623 | 0.9012 | 0.0149 | 0.1236 | 0.3681 | 0.035 | 0.01 (0, 0.05) | 0 (0, 0) | 0 (0, 0.05) | 0 (0, 0) |
| U00004418 | 0.0008 | 0.6232 | 0.0073 | 0.0001 | 0.2006 | 0.0016 | 0.15 (0.13, 0.3) | 0 (0, 0.02) | 0.16 (0.12, 0.69) | 0.02 (0, 0.07) |
| U00208153 | 0.0002 | 0.0012 | 0.0002 | 0.3681 | na | 0.3681 | 0.21 (0.09, 0.35) | 0 (0, 0) | 0 (0, 0) | 0 (0, 0) |
| U00000374 | 0.0002 | 0.3681 | 0.0002 | 0.0005 | 0.8798 | 0.0005 | 0 (0, 0) | 0.13 (0.09, 0.22) | 0 (0, 0) | 0.19 (0.09, 0.46) |
| U00030870 | 0.0008 | 0.1094 | 0.0019 | 0.035 | 0.3681 | 0.1236 | 0.17 (0.07, 0.22) | 0 (0, 0) | 0 (0, 0.11) | 0 (0, 0) |
| U00837797 | 0.0022 | 0.0022 | 0.0022 | na | na | na | 0.04 (0.01, 0.21) | 0 (0, 0) | 0 (0, 0) | 0 (0, 0) |
| U00449804 | 0.0001 | 0.0001 | 0.0001 | 0.3681 | na | 0.3681 | 0.16 (0.06, 0.25) | 0 (0, 0) | 0 (0, 0) | 0 (0, 0) |
| U00436332 | 0.006 | 0.0296 | 0.006 | 0.1681 | na | 0.1681 | 0.07 (0, 0.1) | 0 (0, 0) | 0 (0, 0) | 0 (0, 0) |
| U00003435 | 0.0001 | 0.0535 | 0.0001 | 0.0022 | na | 0.0022 | 0.21 (0.16, 0.29) | 0 (0, 0) | 0.07 (0.01, 0.1) | 0 (0, 0) |
| U00198673 | 0.035 | 0.6898 | 0.035 | 0.0779 | na | 0.0779 | 0 (0, 0.09) | 0 (0, 0) | 0 (0, 0.06) | 0 (0, 0) |
| U00002720 | 0.0022 | 0.0063 | 0.0022 | 0.3681 | na | 0.3681 | 0.04 (0.01, 0.05) | 0 (0, 0) | 0 (0, 0) | 0 (0, 0) |
| U00195707 | 0.006 | 0.8452 | 0.006 | 0.006 | na | 0.006 | 0.09 (0, 0.22) | 0 (0, 0) | 0.07 (0, 0.12) | 0 (0, 0) |
| U00002686 | 0.0008 | 0.2062 | 0.0008 | 0.006 | na | 0.006 | 0.13 (0.08, 0.22) | 0 (0, 0) | 0.03 (0, 0.13) | 0 (0, 0) |
| U00008959 | 0.0002 | 0.0004 | 0.0002 | 0.3681 | na | 0.3681 | 0.07 (0.04, 0.22) | 0 (0, 0) | 0 (0, 0) | 0 (0, 0) |
| U00034351 | 0.0002 | 0.0277 | 0.0002 | 0.035 | na | 0.035 | 0.19 (0.14, 0.27) | 0 (0, 0) | 0 (0, 0.06) | 0 (0, 0) |
| U00437009 | 0.0009 | 0.0004 | 0.0002 | 1 | 0.3681 | 0.3681 | 0.07 (0.05, 0.1) | 0 (0, 0) | 0 (0, 0) | 0 (0, 0) |
| U00032486 | 0.0022 | 0.0082 | 0.0022 | 0.3681 | na | 0.3681 | 0.11 (0.01, 0.29) | 0 (0, 0) | 0 (0, 0) | 0 (0, 0) |
| U00000999 | 0.0001 | 0.0007 | 0.0001 | 0.1236 | 1 | 0.1012 | 0.42 (0.25, 0.61) | 0 (0, 0) | 0 (0, 0.09) | 0 (0, 0) |
| U00002201 | 0.0001 | 0.0003 | 0.0001 | 0.3681 | na | 0.3681 | 0.23 (0.19, 0.33) | 0 (0, 0) | 0 (0, 0) | 0 (0, 0) |
| U00001891 | 0.0072 | 0.0033 | 0.0008 | 1 | 0.3681 | 0.3681 | 0.09 (0.05, 0.18) | 0 (0, 0) | 0 (0, 0) | 0 (0, 0) |
| U00803665 | 0.0022 | 0.0022 | 0.0022 | na | na | na | 0.12 (0.01, 0.15) | 0 (0, 0) | 0 (0, 0) | 0 (0, 0) |
| U00027111 | 0.0001 | 0.0866 | 0.0001 | 0.0149 | na | 0.0149 | 0.32 (0.05, 0.48) | 0 (0, 0) | 0.03 (0, 0.19) | 0 (0, 0) |
| U00454157 | 0.0009 | 0.0002 | 0.0002 | 0.3681 | 0.3681 | na | 0.14 (0.05, 0.32) | 0 (0, 0) | 0 (0, 0) | 0 (0, 0) |
| U00080174 | 0.035 | 0.8311 | 0.035 | 0.035 | na | 0.035 | 0 (0, 0.04) | 0 (0, 0) | 0 (0, 0.04) | 0 (0, 0) |
| U00003296 | 0.1681 | na | 0.0022 | 0.1681 | 0.0146 | 0.0022 | 0 (0, 0) | 0 (0, 0) | 0 (0, 0) | 0.04 (0.01, 0.11) |
| U00803302 | 0.0001 | 0.0004 | 0.0001 | 0.0779 | na | 0.0779 | 0.09 (0.05, 0.21) | 0 (0, 0) | 0 (0, 0.02) | 0 (0, 0) |
| U00032208 | 0.0022 | 0.0105 | 0.0022 | 0.3681 | na | 0.3681 | 0.06 (0.01, 0.14) | 0 (0, 0) | 0 (0, 0) | 0 (0, 0) |
| U00001116 | 0.0001 | 0.9698 | 0.0001 | 0.0001 | 0.5036 | 0.0001 | 0.83 (0.43, 1.04) | 0 (0, 0) | 0.6 (0.34, 1.27) | 0 (0, 0) |
| U00807704 | 0.0008 | 0.0522 | 0.0008 | 0.0779 | na | 0.0779 | 0.05 (0.04, 0.07) | 0 (0, 0) | 0 (0, 0.03) | 0 (0, 0) |
| U00000272 | 0.0004 | 0.6776 | 0.0001 | 0.0034 | 0.5447 | 0.0013 | 0.57 (0.29, 0.99) | 0.03 (0, 0.06) | 0.65 (0.25, 0.79) | 0 (0, 0.06) |
| U00027329 | 0.1681 | na | 0.006 | 0.1681 | 0.045 | 0.006 | 0 (0, 0) | 0 (0, 0) | 0 (0, 0) | 0.04 (0, 0.11) |
| U00003420 | 0.3681 | 0.3681 | 0.0022 | 1 | 0.0133 | 0.0133 | 0 (0, 0) | 0 (0, 0) | 0 (0, 0) | 0.12 (0.02, 0.22) |
| U00436238 | 0.5842 | 0.2343 | 0.0004 | 0.5848 | 0.0038 | 0.0155 | 0 (0, 0) | 0 (0, 0) | 0 (0, 0.06) | 0.12 (0.09, 0.14) |
| U00805277 | 0.0008 | 0.5656 | 0.0008 | 0.006 | na | 0.006 | 0.09 (0.04, 0.13) | 0 (0, 0) | 0.06 (0, 0.09) | 0 (0, 0) |
| U01136238 | 0.0022 | 0.0022 | 0.0022 | na | na | na | 0.05 (0.01, 0.08) | 0 (0, 0) | 0 (0, 0) | 0 (0, 0) |
| U00848639 | 0.0022 | 0.1062 | 0.0022 | 0.0779 | na | 0.0779 | 0.07 (0.01, 0.18) | 0 (0, 0) | 0 (0, 0.05) | 0 (0, 0) |
| U00439751 | 0.0022 | 0.3681 | 0.006 | 0.0063 | 0.7869 | 0.0187 | 0 (0, 0) | 0.04 (0.01, 0.06) | 0 (0, 0) | 0.03 (0, 0.06) |
| U00004741 | 0.3681 | na | 0.0149 | 0.3681 | 0.0934 | 0.0149 | 0 (0, 0) | 0 (0, 0) | 0 (0, 0) | 0.01 (0, 0.04) |
| U00004249 | 0.0002 | 0.0036 | 0.0001 | 0.0337 | 0.1206 | 0.0041 | 2.83 (1.67, 3.73) | 0.02 (0, 0.16) | 0.59 (0.07, 1.28) | 0 (0, 0) |
| U01160251 | 0.5036 | 0.3681 | 0.0001 | 0.1681 | 0.0002 | 0.0001 | 0 (0, 0) | 0 (0, 0) | 0 (0, 0) | 0.28 (0.23, 0.35) |
| U01166410 | 0.5036 | 0.5036 | 0.0001 | 0.8712 | 0.0016 | 0.0007 | 0 (0, 0) | 0 (0, 0) | 0 (0, 0) | 0.13 (0.08, 0.3) |
| U01169117 | 0.3681 | 0.3681 | 0.0008 | 1 | 0.0091 | 0.0072 | 0 (0, 0) | 0 (0, 0) | 0 (0, 0) | 0.03 (0.02, 0.04) |
| U01180557 | na | na | 0.006 | na | 0.006 | 0.006 | 0 (0, 0) | 0 (0, 0) | 0 (0, 0) | 0.02 (0, 0.03) |
| U00804007 | 0.0837 | 0.1984 | 0.0001 | 0.5182 | 0.0005 | 0.0002 | 0 (0, 0) | 0.06 (0, 0.21) | 0.01 (0, 0.07) | 1.29 (0.93, 1.47) |
| U01143008 | 0.1681 | na | 0.006 | 0.1681 | 0.0962 | 0.006 | 0 (0, 0) | 0 (0, 0) | 0 (0, 0) | 0.02 (0, 0.06) |
| U01145549 | 0.1681 | 0.3681 | 0.0001 | 0.5842 | 0.0044 | 0.0013 | 0 (0, 0) | 0 (0, 0) | 0 (0, 0) | 0.05 (0.04, 0.06) |
| U00808391 | 0.2343 | 0.3681 | 0.0004 | 0.0779 | 0.0039 | 0.0002 | 0 (0, 0) | 0 (0, 0.07) | 0 (0, 0) | 0.2 (0.19, 0.24) |
| U01144224 | 0.3681 | na | 0.006 | 0.3681 | 0.0298 | 0.006 | 0 (0, 0) | 0 (0, 0) | 0 (0, 0) | 0.02 (0, 0.04) |
| U01214804 | na | na | 0.0022 | na | 0.0022 | 0.0022 | 0 (0, 0) | 0 (0, 0) | 0 (0, 0) | 0.02 (0.01, 0.04) |
| U01173110 | 0.1681 | na | 0.0008 | 0.1681 | 0.0138 | 0.0008 | 0 (0, 0) | 0 (0, 0) | 0 (0, 0) | 0.05 (0.02, 0.13) |
| U00305232 | 0.1433 | 0.376 | 0.0001 | 0.4818 | 0.0005 | 0.0002 | 0 (0, 0) | 0.03 (0, 0.16) | 0 (0, 0.05) | 1.28 (0.98, 1.5) |
| U00043735 | 0.0503 | 0.1236 | 0.0001 | 0.5907 | 0.0016 | 0.0001 | 0 (0, 0) | 0.02 (0, 0.06) | 0 (0, 0.05) | 0.19 (0.15, 0.37) |
| U00000409 | 0.1826 | 0.0187 | 0.0002 | 0.3068 | 0.0002 | 0.0002 | 0.05 (0.01, 0.15) | 0.26 (0.05, 0.52) | 0.35 (0.25, 0.9) | 2.73 (1.89, 2.96) |
| U00029718 | na | na | 0.006 | na | 0.006 | 0.006 | 0 (0, 0) | 0 (0, 0) | 0 (0, 0) | 0.04 (0, 0.08) |
| U00007185 | 0.5842 | 0.0403 | 0.0002 | 0.1984 | 0.0007 | 0.0054 | 0 (0, 0) | 0 (0, 0) | 0.04 (0, 0.08) | 0.28 (0.12, 0.39) |
| U00011248 | 0.7329 | 0.5182 | 0.0002 | 0.348 | 0.0002 | 0.0012 | 0.01 (0, 0.05) | 0 (0, 0.07) | 0.02 (0, 0.15) | 0.65 (0.58, 0.79) |
| U01156257 | 0.1681 | na | 0.0149 | 0.1681 | 0.1692 | 0.0149 | 0 (0, 0) | 0 (0, 0) | 0 (0, 0) | 0.02 (0, 0.08) |
| U00808203 | 0.1681 | 0.3681 | 0.0002 | 0.5036 | 0.0038 | 0.0004 | 0 (0, 0) | 0 (0, 0) | 0 (0, 0) | 0.19 (0.16, 0.27) |
| U00003073 | 0.1681 | na | 0.0022 | 0.1681 | 0.0283 | 0.0022 | 0 (0, 0) | 0 (0, 0) | 0 (0, 0) | 0.07 (0.02, 0.12) |
| U00012096 | 0.0022 | 0.0022 | 0.0022 | na | na | na | 0.05 (0.01, 0.08) | 0 (0, 0) | 0 (0, 0) | 0 (0, 0) |
| U01152122 | 0.0022 | 0.0022 | 0.0022 | na | na | na | 0.24 (0.04, 0.53) | 0 (0, 0) | 0 (0, 0) | 0 (0, 0) |
| U01123199 | 0.9097 | 0.3073 | 0.0001 | 0.1604 | 0.0014 | 0.0014 | 0.17 (0.06, 0.45) | 0.22 (0.12, 0.37) | 0.46 (0.21, 0.74) | 0 (0, 0) |
| U00000235 | 0.244 | 0.6704 | 0.0001 | 0.1799 | 0.0011 | 0.0001 | 0 (0, 0) | 0 (0, 0.11) | 0 (0, 0) | 1.52 (0.91, 2.48) |
| U00000737 | 0.0022 | 0.0239 | 0.0022 | 0.035 | na | 0.035 | 0.13 (0.03, 0.38) | 0 (0, 0) | 0 (0, 0.05) | 0 (0, 0) |
| U00822040 | 0.0022 | 0.0752 | 0.0049 | 0.1681 | 0.3681 | 0.5036 | 0.04 (0.01, 0.09) | 0 (0, 0) | 0 (0, 0) | 0 (0, 0) |
